# Magnetic Resonance Imaging‐Based Quantification of Endosomal Escape Using Iron Oxide Nanoparticle‐Loaded Lipid Nanoparticles

**DOI:** 10.1002/adhm.202503055

**Published:** 2025-08-05

**Authors:** Somin Lee, Jeongbin Park, Han Na Jung, Shengjun Li, Zhijun Lin, Hyung‐Jun Im

**Affiliations:** ^1^ Department of Molecular Medicine and Biopharmaceutical Sciences Graduate School of Convergence Science and Technology Seoul National University Seoul 03080 Republic of Korea; ^2^ Program in Biomedical Sciences University of Michigan Ann Arbor MI 48109 USA; ^3^ Department of Applied Bioengineering Graduate School of Convergence Science and Technology Seoul National University Seoul 08826 Republic of Korea; ^4^ Portrai, Inc. Seoul 03136 Republic of Korea; ^5^ Cancer Research Institute Seoul National University Seoul 03080 Republic of Korea; ^6^ Research Institute for Convergence Science Seoul National University Seoul 08826 Republic of Korea

**Keywords:** drug delivery, endosomal escape, in vivo imaging, iron oxide nanoparticles, lipid nanoparticles, magnetic resonance imaging

## Abstract

Lipid nanoparticles (LNPs) are considered promising and advanced nucleic acid‐based therapeutic delivery platforms. The therapeutic efficacy of LNP‐based drugs depends heavily on endosomal escape. However, few methods are available for quantifying the efficiency of endosomal escape in vivo. Herein, a novel method for quantifying endosomal escape efficiency using magnetic resonance imaging (MRI) is presented. In this method, ultrasmall iron oxide nanoparticles (IONPs) are synthesized and incorporated into LNPs, generating IONPs‐loaded LNPs (IO@LNPs). After cellular internalization of IO@LNPs, the *R*_2_ relaxation rate is reduced over time, suggesting the dispersal of free IONPs owing to endosomal escape. Data from electron microscopy further corroborated this finding, showing a strong correlation between the *R*_2_ value and the number of intracellular endosomes harboring intact IO@LNPs. In vivo MRI from mice demonstrated a gradual decrease in *R*_2_ signals at the tissue site where IO@LNPs are injected, indicating the potential application of the proposed method in vivo. These findings can advance LNP‐based nucleic acid delivery research by enhancing the understanding of endosomal escape in vivo.

## Graphical abstract

The therapeutic efficacy of lipid nanoparticle(LNP)‐based drugs depends on endosomal escape. However, few methods are available for quantifying the efficiency of endosomal escape in vivo. Herein, a novel method for quantifying the endosomal escape efficiency index using magnetic resonance imaging is introduced. This technique can facilitate advancements in LNP‐based nucleic acid delivery by enhancing the understanding of endosomal escape in vivo.

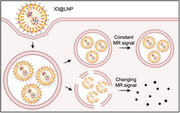

## 1 Introduction

**Figure 1:**
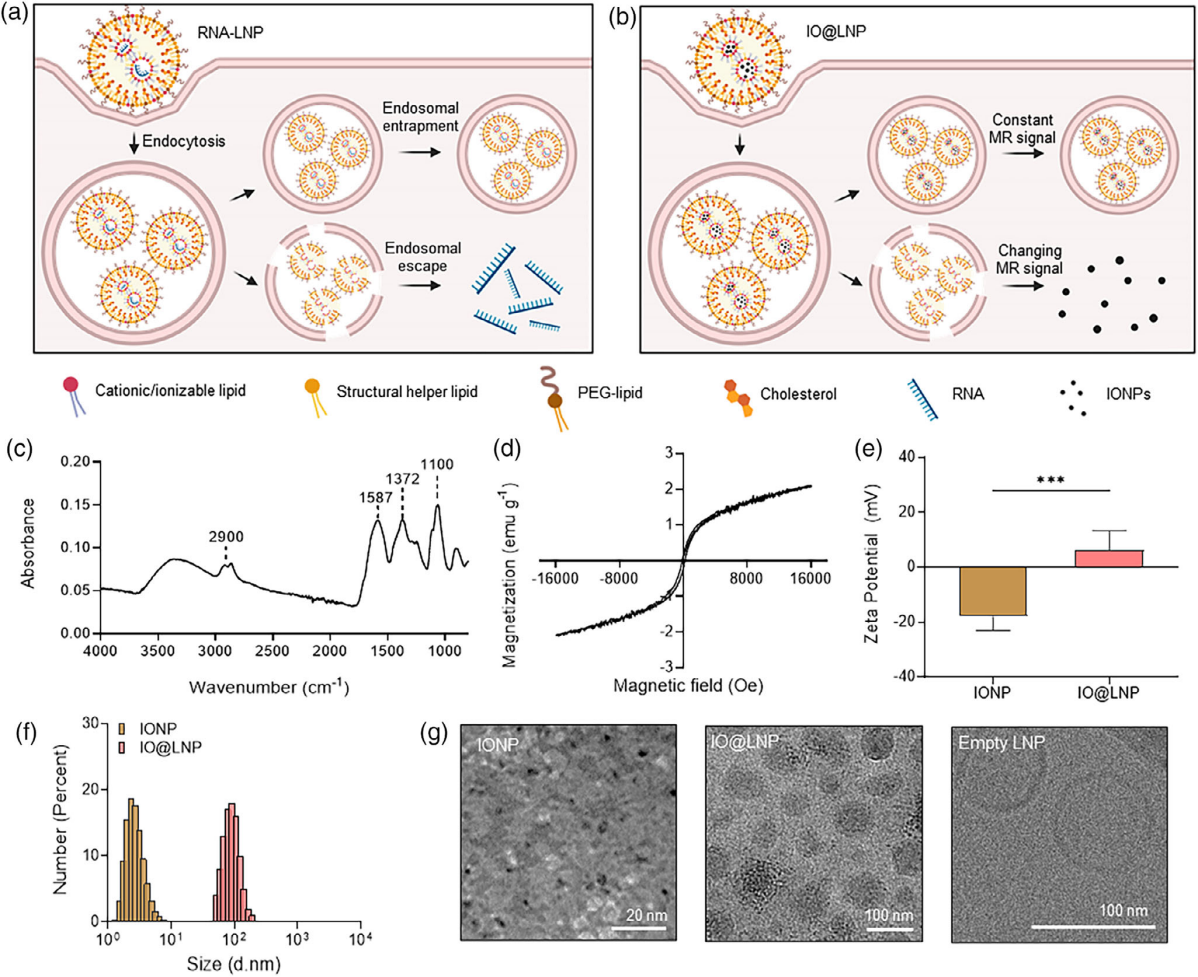
Endosomal escape and physicochemical characteristics of IONPs and IO@LNPs. a) Schematic illustration of endosomal escape. RNA molecules in LNPs can only exert their therapeutic efficacy after escaping the endosomes. b) A novel method for evaluating the endosomal escape efficiency index using IONPs and MRI. Like RNA molecules, anionic IONPs can be incorporated with cationic/ionizable lipids. IONPs loaded in LNPs are dispersed into the cytosol via endosomal escape, leading to a decrease in the *R*_2_ signal. c) A FTIR spectrum of IONPs. d) Normalized field‐dependent magnetization (M–H) curve of IONPs determined using a VSM at 300 K. The normalized saturation moment of IONPs was ≈3 emu g^−1^. e,f) Comparison of the zeta potential (e) and size distribution (f) of IONPs and IO@LNPs. Data presented as mean ± SD, *n* = 5, *p*‐values are calculated using T‐test, ^***^ *p* < 0.001. g) Representative microscopic images of IONPs, IO@LNPs, and empty LNPs. The nanoparticles exhibit a round shape and uniform size. Tiny black dots inside IO@LNPs indicate the presence of IONPs.

Lipid nanoparticles (LNPs) have recently emerged as an optimal platform for delivering nucleic acid molecules, surpassing traditional drug delivery systems (DDS), as demonstrated by the success of LNP‐based messenger RNA (mRNA) vaccines during the coronavirus disease 2019 (COVID‐19) pandemic.^[^ ^1^ ^]^ Possible components of RNA‐loaded LNPs (RNA‐LNPs) include cationic/ionizable lipids, structural helper lipids (phospholipids), polyethylene glycol (PEG)‐anchored lipids, cholesterol, and anionic RNA cargos (**Figure** **1**a). The cargo can be small interfering RNA (siRNA), microRNA (miRNA), circular RNA (circRNA), and mRNA.^[^ ^2^, ^3^, ^4^, ^5^ ^]^ RNAs of different structures and sizes integrate into LNPs through the electrostatic attraction between anionic RNA molecules and cationic or ionizable lipids, forming RNA‐LNPs that are generally taken up by cells through endocytosis and reside within the cell endosomes.

Among the various biological barriers that can hinder the therapeutic efficacy of RNA‐based drugs, the endosome is the primary and most challenging intracellular barrier.^[^ ^6^, ^7^ ^]^ Currently, RNA‐LNP‐based therapeutics have overcome the endosomal barrier by leveraging on the cationic or ionizable lipids within the LNPs. These lipids interact with the anionic endosomal membranes, facilitating the release of RNA molecules into the cytoplasm through a process referred to as endosomal escape.^[^ ^8^, ^9^, ^10^ ^]^ In addition, phospholipids containing unsaturated bonds—such as 1,2‐dioleoyl‐sn‐glycero‐3‐phosphoethanolamine (DOPE)—can promote endosomal membrane destabilization through hexagonal phase transition, further contributing to the efficiency of endosomal escape.^[^ ^11^, ^12^ ^]^

Previous studies have established a direct correlation between successful endosomal escape and the therapeutic efficacy of RNA‐based drugs.^[^ ^1^, ^13^ ^]^ However, current strategies for promoting endosomal escape remain largely ineffective, with 1–2% of administered RNA reaching the cytoplasm.^[^ ^14^, ^15^ ^]^ Consequently, recent efforts have concentrated on improving endosomal escape,^[^ ^16^, ^17^ ^]^ necessitating the development of innovative techniques for accurately measuring the escape efficiency and identifying effective LNP‐based delivery platforms. Traditional methods for assessing endosomal escape, including fluorescent labeling and transfection assays, are limited.^[^ ^18^, ^19^, ^20^, ^21^, ^22^ ^]^ Commonly used assays that measure RNA transfection efficiency using expression of reporter proteins are indirect methods.^[^ ^7^ ^]^ These methods are predominantly restricted to microscopic analysis and unsuitable for in vivo studies, hindering the comprehensive analysis of LNP‐facilitated endosomal escape of RNA.

Herein, we present a novel method for evaluating the efficiency index of endosomal escape using magnetic resonance imaging (MRI), one of the most widely used in vivo imaging modalities. Our methodology integrates MRI with iron oxide nanoparticles (IONPs), with the latter serving as an MRI contrast agent for amplifying the magnetic resonance (MR) signals. Consequently, we synthesized IONP‐loaded LNPs (IO@LNPs) and performed the MRI scan in multicellular and in vivo settings. By analyzing changes in MRI signals, we estimated the efficiency index of endosomal escape.

We hypothesized that IONPs clustered within LNPs would generate a distinct MRI signal compared with those of the cytoplasmic‐dispersed IONPs following endosomal escape (Figure 1b). Consequently, changes in the MRI signal may serve as an effective metric for determining the endosomal escape efficiency index.

## 2 Results and Discussion

### 2.1 Characterization of IONPs and IO@LNPs

Ultrasmall IONPs (< 8 nm) have been reported to penetrate the glomerular filtration barrier of the kidneys, resulting in high renal clearance without toxicity.^[^ ^23^, ^24^, ^25^ ^]^ In a previous study, citrate‐stabilized ultrasmall IONPs have been synthesized using a solvothermal method without surface coatings such as PEG.^[^ ^26^ ^]^ The surface of IONPs is generally modified with a polymer coating for prolonged blood circulation and long‐term colloidal stability in cells.^[^ ^27^, ^28^ ^]^ However, unmodified IONPs carry a more negative surface charge, making their encapsulation in LNPs easier.^[^ ^29^ ^]^ This process is similar to the integration of nucleic acids with cationic/ionizable lipids. Hence, bare IONPs were selected in this study over conventional IONPs.

The peaks observed in the Fourier‐transform infrared (FTIR) spectrum confirmed the successful synthesis of the IONPs (Figure 1c).^[^ ^26^, ^29^ ^]^ The peaks at 1587 and 1372 cm^−1^ corresponded to the stretching vibrations of conjugated C═O and C─O bonds, whereas those at ≈2900 and 1100 cm^−1^ indicated the stretching vibrations of ─CH_2_ and ─OH groups of sodium citrate, respectively. The presence of these peaks assigned to key functional groups suggested that the surface of IONPs was successfully stabilized with citrate. A vibrating sample magnetometer (VSM) test was conducted to measure the magnetic properties of the IONPs, which are pertinent to their contrast effect in MRI (Figure 1d). The normalized saturation moment of the IONPs was ≈3 emu g^−1^, consistent with the values reported in the literature.^[^ ^26^, ^29^ ^]^ In addition, the ability of IONPs to respond sensitively to an external magnetic field in the hysteresis loop indicated that these nanoparticles are either ferromagnetic or superparamagnetic. However, the near‐zero coercivity implied that the IONPs are not ferromagnetic but solely superparamagnetic.

IO@LNPs (Fe:lipid = 1/20 w/w) of a homogenous size were prepared with 1,2‐di‐O‐octadecenyl‐3‐trimethylammonium propane (DOTMA; cationic lipid), 1,2‐distearoyl‐sn‐glycero‐3‐phosphocholine (DSPC; structural helper phospholipid), 1,2‐distearoyl‐sn‐glycero‐3‐phosphoethanolamine‐N‐[methoxy(polyethylene glycol)‐5000] (mPEG‐DSPE; PEGylated lipid), and cholesterol using a fluidic‐based mixture. This LNP formulation was validated by encapsulating mRNAs. We first synthesized in vitro transcription (IVT) mRNA encoding tdTomato fluorescence protein (Table S1 and Figure S1a,b, Supporting Information). The IVT tdTomato mRNA successfully induced the fluorescent signal following lipofectamine‐based transfection (Figure S1c, Supporting Information). Subsequently, the mRNAs were encapsulated into LNP and transfected into cells (Figure S2, Supporting Information). The mRNAs delivered by the LNP were successfully expressed as fluorescence proteins, as confirmed by flow cytometry analysis. Dose‐dependent enhancement of protein expression was observed with increasing amounts of mRNA‐LNPs.

IO@LNP was prepared by incorporating IONPs into the formulation, using the same lipid composition as the tdTomato mRNA‐LNP. The zeta potential (surface charge) of IO@LNPs was 6.11 ± 7.18 mV (mean ± SD), which was more positive than that of IONPs (−18.0 ± 5.18 mV) (*P* = 0.0003)(Figure 1e). The size of IONPs, measured by dynamic light scattering, was 2.289 ± 0.641 nm (mean ± SD) with a polydispersity index (PDI) of 0.235 (Figure 1f). In comparison, these values for IO@LNPs was 93.13 ± 30.66 nm with PDI of 0.166. The surface charge and size distribution results suggested that the negatively charged IONPs interacted with the positively charged lipids in the LNPs, possibly reducing the effective number of cationic lipids on the surface of IO@LNPs.^[^ ^30^ ^]^

The structures of IONPs and LNPs were observed using transmission electron microscopy (TEM) and cryo‐electron microscopy (cryo‐EM), respectively. The images in Figure 1g show the morphologies of IONPs, IO@LNPs, and empty LNPs. The negative‐staining TEM images reveal that the IONPs are ultrasmall in size and uniformly dispersed. The cryo‐EM images of IO@LNPs demonstrate successful integration of IONPs into LNPs, forming a compact core that is clearly distinguishable from the vacant core observed in empty LNPs. Because empty LNPs lacked negatively charged molecules (e.g., IONP or mRNA) to interact with the positively charged lipids, they took on a liposome form. In addition, electrophoresis analysis based on polarity differences, as indicated by the zeta potential measurements, further confirmed the successful incorporation of IONPs into the LNPs (Figure S3, Supporting Information). Furthermore, stability tests were conducted on IONPs and IO@LNPs. The IONPs remained stable for 7 h (Figure S4a, Supporting Information). The size and PDI of IO@LNPs remained stable for 7 days (Figure S4b, Supporting Information). The hydrodynamic diameters of IO@LNPs, measured by dynamic light scattering (DLS) after incubation at endosomal and lysosomal pH values (6.5, 5.5, and 4.5), remained stable for more than 10 days (Figure S4c, Supporting Information). Moreover, the size distributions at three acidic conditions were highly similar for up to 273 h (Figure S4d, Supporting Information).

### 2.2 MR Properties of IONPs and IO@LNPs

In MRI technology, the *R*_2_ relaxation rate is the reciprocal of the transverse relaxation time (*T*_2_), which represents the progression of the transverse component of proton magnetization from 100% (pulse flip) to 37%. Similarly, the *R*_1_ relaxation rate is the reciprocal of the longitudinal relaxation time (*T*_1_), which represents the progression of the longitudinal component of proton magnetization from 0% (pulse flip) to 63%. Graphical MRI data obtained in the time domain can be converted to images expressed as *R*_1_ or *R*_2_ values in the frequency domain. The *r*_1_ and *r*_2_ relaxivities represent the changes in the *R*_1_ and *R*_2_ values divided by the changes in iron concentration, respectively. In principle, the *r*_1_ and *r*_2_ relaxivities are constant regardless of iron concentration. In this study, the units of *r*_1_ and *r*_2_ are assigned as s^−1^[mg mL^−1^ Fe]^−1^.

To examine the MR signals produced by the lipid components of LNPs, the MR signal of empty LNPs was analyzed (Figure S5, Supporting Information). The signal from the empty LNPs (*r*_1_ = −0.017 and *r*_2_ = 0.031 s^−1^ [mg mL^−1^ lipids]^−1^) remained consistently low, with only a marginal deviation from 0. Thus, we regarded the MR signals from the lipid components of LNPs as negligible. The IO@LNP samples were prepared at varying Fe‐to‐lipid ratios (w/w). In the same batch experiment, the *r*_2_ value showed no significant difference between IO@LNPs with lower and higher Fe concentrations (*r*_2_ = 26.3 for 1/40 w/w and *r*_2_ = 27.3 for 1/20 w/w, respectively) (Figure S6, Supporting Information). Thus, IO@LNPs with a Fe‐to‐lipid ratio of 1/20 w/w were used in the following experiments.

**Figure 2:**
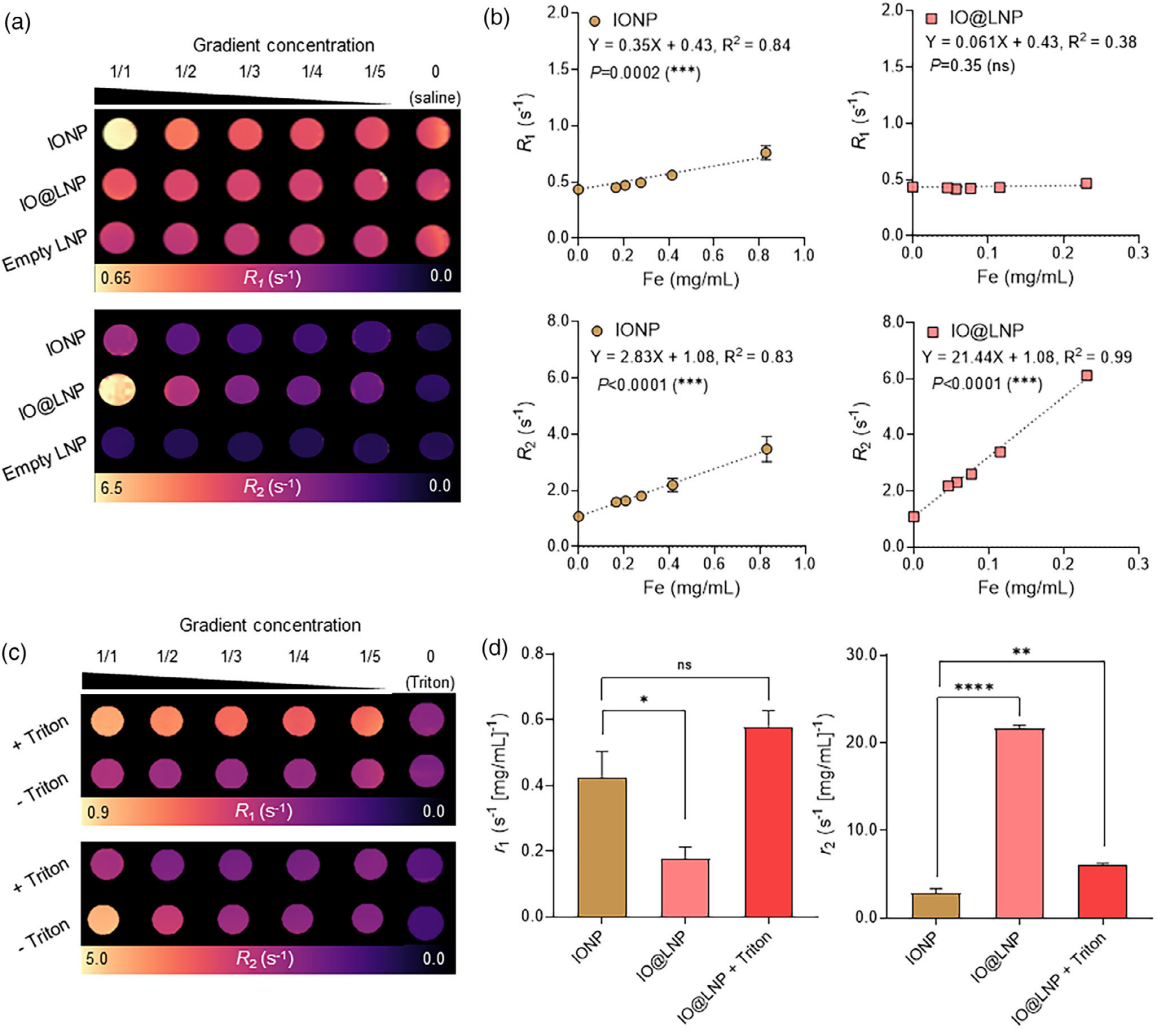
MRI phantom test of IONPs and IO@LNPs. a) Representative MRI phantom mapping images of IONPs, IO@LNPs, and empty LNPs. The samples were prepared by serial dilution from 1/1 (0 mg mL^−1^ Fe and 4.33 mg mL^−1^ lipid for empty LNP samples; 0.23 065 mg mL^−1^ Fe and 4.33 mg mL^−1^ lipid for IO@LNP samples; 0.82 955 mg mL^−1^ Fe and 0 mg mL^−1^ lipid for IONP samples) to 1/5. All samples were dissolved in saline. b) *R*_1_ and *R*_2_ graphs acquired from MRI phantom maps in (a). Data presented as mean ± SEM, *n* = 3, *p*‐values are calculated using F‐test, ^***^ *p* < 0.001. c) Representative MRI phantom mapping images of IO@LNP with and without treatment of Triton X‐100, which is a detergent used to dissolve LNPs. The images were obtained by serial dilution from 1/1 (0.13 786 mg mL^−1^ Fe and 2.59 mg mL^−1^ lipid) to 1/5. Triton X‐100 had minimal effect on the IONPs. d) Comparison of *r*_1_ and *r*_2_ between IONPs and IO@LNPs with or without Triton X‐100). Data presented as mean ± SEM, *n* = 3, ^*^ *p* < 0.05, ^**^ *p* < 0.01, and ^****^ *p* < 0.0001.

Subsequently, MRI phantom maps were obtained using varying concentrations of IONPs to acquire MR signals from both free IONPs and IO@LNPs. The map images show that while IONPs (*r*_1_ = 0.35) and IO@LNPs (*r*_1_ = 0.061) exhibited detectable *R*_1_ signals, their *R*_2_ values were 8‐ and 351‐fold more prominent, respectively (*r*_2_ = 2.83 for IONPs and *r*_2_ = 21.44 for IO@LNPs) (**Figure** **2**a,b). These results demonstrate that IONPs and IO@LNPs function as *T*_2_ contrast agents (α value for IONP = 8.10 > threshold α value = 5).^[^ ^31^, ^32^ ^]^ In addition, IO@LNPs exhibited lower *r*_1_ and higher *r*_2_ values compared with those of dispersed IONPs. These findings are consistent with the fundamental principles of MR. The *R*_1_ relaxation rate reflects an indicator of the speed by which the protons in water return to equilibrium after excitation by radio frequency (RF) energy during MRI scanning. Dispersed IONPs, due to their greater mobility, therefore facilitated more efficient dissipation of RF energy, leading to an increase in the *R*_1_ value compared with that of IO@LNPs. In contrast, the *R*_2_ relaxation rate increases as the local magnetic field inhomogeneities increase in proportion to the square of the magnetic moment.^[^ ^27^, ^33^ ^]^ When the IONPs were clustered within the LNPs, their magnetic moments aligned in an additive manner, thereby increasing the *R*_2_ value. Thus, IO@LNPs had a larger *R*_2_ value compared with that of the dispersed IONPs. These properties were also demonstrated in a previous study that compared the relaxation rates of ultrasmall IONPs and their clusters.^[^ ^34^ ^]^

To confirm the reduction in *R*_2_ value resulting from the dispersion of IONPs from LNPs, IO@LNPs were treated with the detergent Triton X‐100 to induce the dissociation of LNPs and subsequent release of IONP (Figure 2c). The Triton X‐100–treated IO@LNP (*r*_1_ = 0.58 and *r*_2_ = 6.14) exhibited a 3.2‐fold higher *r*_1_ but 3.5‐fold lower *r*_2_ value compared to Triton–untreated IO@LNPs (*r*_1_ = 0.18 and *r*_2_ = 21.65) (Figure 2d). These results indicate that detergent treatment led to the dissociation of IO@LNPs and the release of IONPs. Furthermore, IONPs dispersed from LNPs showed MR properties comparable to those of the original free IONPs (*r*_1_ = 0.42 and *r*_2_ = 2.87), supporting the feasibility of using this strategy to detect endosomal escape.

### 2.3 In Vitro MRI for Evaluating Endosomal Escape

To determine the observation window for the endosomal escape of IO@LNPs in vitro, the temporal trends of LNP uptake in cells were assessed using IO@LNPs labeled with DiD, a fluorescence dye, (IO@LNP/DiD) (Figure S7, Supporting Information). The cellular uptake of IO@LNP/DiD was observed using confocal laser scanning microscopy (CLSM). Cells were treated with the same amount of IO@LNP/DiD and washed after different treatment time points (Figure S8a, Supporting Information). As expected, no discernible intracellular uptake of LNPs was observed at 0 min. Therefore, to investigate the internalization dynamics of the LNPs at time points starting from 20 min, we analyzed the fluorescence intensity profiles of individual laser channels (blue for nuclei and red for LNPs) (Figure S8c, Supporting Information). The relative distance between the nuclear center and each LNP‐associated red pixel along the cross‐sectional line was 1.3‐fold greater in cells treated with IO@LNPs for 20 min than in those treated for 40 min (*p* = 0.0001) (Figure S8d, Supporting Information). These results suggest that by 40 min, the LNPs had migrated toward more intracellular regions. This finding was consistent with the previously reported rapid endosomal trafficking within 2 h.^[^ ^35^ ^]^ Based on this, we decided to observe the process of endosomal escape starting from 40 min.

**Figure 3:**
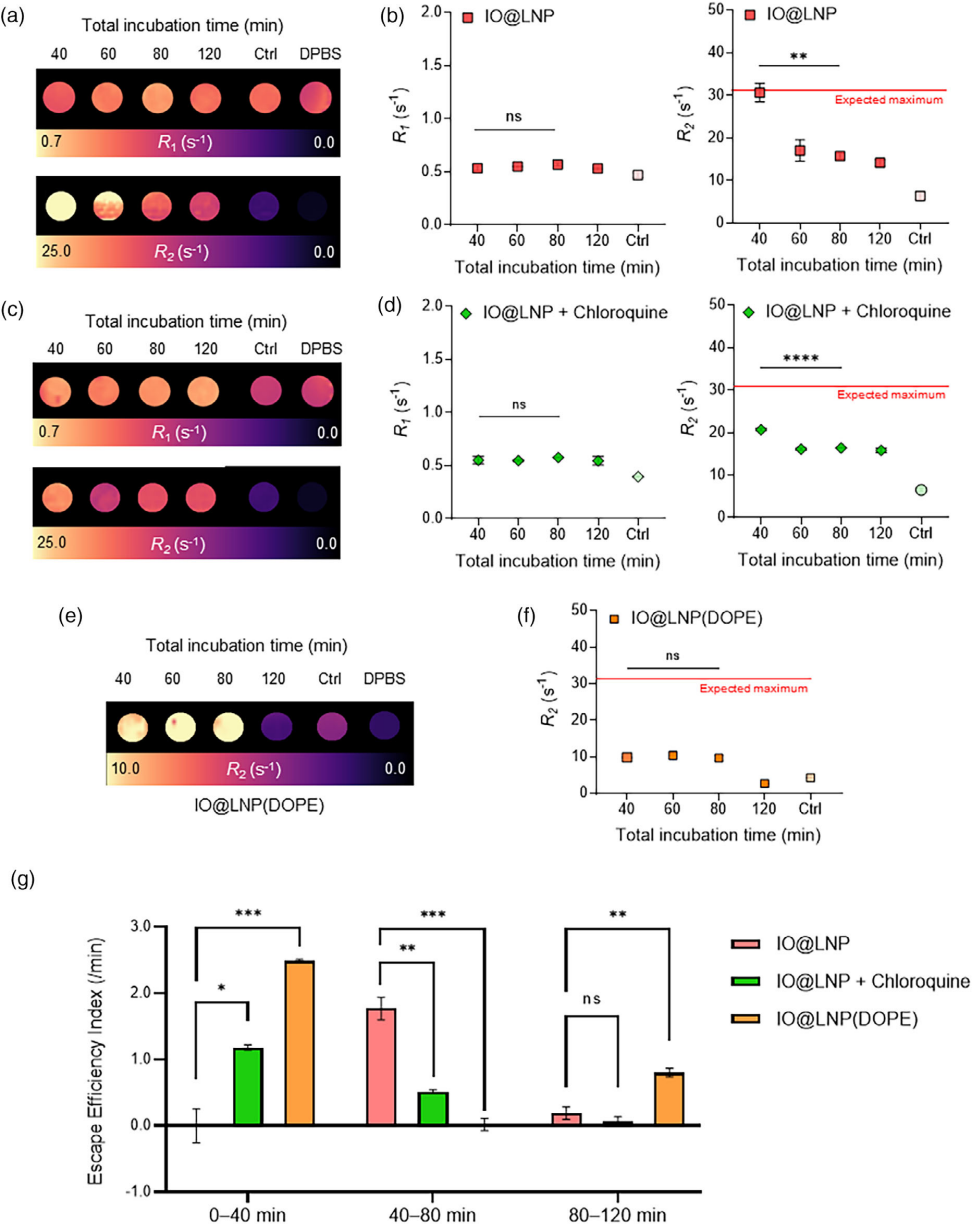
In vitro MRI for evaluating endosomal escape. a,b) Representative in vitro *R*_1_ and *R*_2_ mapping images of IO@LNP‐treated cell pellets at various incubation times (40, 60, 80, and 120 min). The control group (Ctrl) was not treated with IO@LNPs. (b) *R*_1_ and *R*_2_ graphs acquired from in vitro MRI results (a). c) Representative in vitro *R*_1_ and *R*_2_ mapping images of chloroquine‐treated groups. Chloroquine facilitates endosomal escape in cells. d) *R*_1_ and *R*_2_ graphs acquired from in vitro MRI results (c). e) In vitro MRI results for IO@LNP(DOPE). f) *R*_2_ graphs acquired from in vitro MRI results (e). g) Calculation of endosomal escape efficiency index within three intervals according to experimental conditions and LNP formulations. All samples were dissolved in Dulbecco's phosphate‐buffered saline (DPBS). All data presented as mean ± SEM, *n* = 3, *p*‐values are calculated using ANOVA‐test, ^*^ *p* < 0.05, ^**^ *p* < 0.01, ^***^ *p* < 0.001, and ^****^ *p* < 0.0001. The expected maximum values for all *R*_2_ graphs were calculated by multiplying the actual Fe concentration by *r*_2, loaded_ (21.44) and then adding the *R*_2_ value of cells.

To measure the MRI signals of IO@LNP‐treated cells, the cells were incubated with IO@LNPs for 40 min, followed by thorough washing to remove any uninternalized IO@LNPs remaining in the medium. Cells were then incubated for additional time periods (0, 20, 40, and 80 min) to allow endosomal escape (Figure S9, Supporting Information). Cells harvested from each group were fixed with 4% paraformaldehyde, and the resulting cell pellets were prepared for MR signal acquisition. As expected from the minor difference in *r*_1_ values between IO@LNPs and free IONPs (Figure 2a,b), the in vitro *R*_1_ value measured between 40 and 80 min showed no statistically significant difference (**Figure** **3**a,b). The lack of a decrease in *R*_1_ values between 40 and 80 min suggests that there was no loss of IONPs within the cells during this time period. The in vitro MR results showed a negligible increase in *R*_1_ values, alongside a more pronounced decrease in *R*_2_ value from 40 to 80 min (*p* = 0.0032), indicating the occurrence of endosomal escape during this period. This finding supports our decision to prioritize the *R*_2_ signal over the *R*_1_ signal.

To confirm that the changes in MR signals were attributed to endosomal escape, cells were treated with chloroquine, a substance known for enhancing endosomal escape (Figure 3c,d).^[^ ^36^ ^]^ Consistent with the chloroquine‐untreated group (Figure 3a,b), no significant difference in the *R*_1_ value was observed at 40–80 min. However, the chloroquine‐treated group exhibited a 1.5‐fold lower initial *R*_2_ values (*R*_2_ = 20.69 s^−1^) at 40 min than the chloroquine‐untreated group (*R*_2_ = 30.65 s^−1^) at the same time point. This observed decrease suggests that, following IO@LNP treatment, the IONPs promptly escaped from the endosomes into the cytosol, a process facilitated by chloroquine, an enhancer of endosomal escape.

### 2.4 MRI‐Based In Vitro Quantification of Endosomal Escape

Using the MR theory and a straightforward application of the chain rule from calculus, we calculated the efficiency index of endosomal escape in /min (Interpretation of MR Signal and Calculation of Endosomal Escape Efficiency Index in Supporting Information). Specifically, this index represents the rate of change in the fraction of encapsulated IONPs, inferred from the decrease in *R*_2_ relaxivity values. We took the average *R*_2_ values measured at 40 min (30.65 s^−1^ averaging three data: 27.53, 34.83, and 29.60 s^−1^) and 120 min (14.19 s^−1^ averaging three data: 14.74, 15.11, and 12.71 s^−1^), and divided their difference by the elapsed time (80 min; 40–120 min duration) to obtain the rate of change in MR signal per unit time. This rate was then normalized by the iron concentration (1.13 mg mL^−1^ Fe) and the difference in intrinsic *r*_2_ relaxivity between LNP‐loaded (21.44 s^−1^[mg mL^−1^ Fe] ^−1^) and free IONPs (2.83 s^−1^[mg mL^−1^ Fe]^−1^), which reflects the contribution of particle to the MR signal. The final result, scaled by 100, yielded an endosomal escape efficiency index of ≈0.98 min^−1^. Previous studies have consistently confirmed that protein expression in HeLa cells begins ≈1.5 h following RNA‐LNP treatment.^[^ ^14^ ^]^ In addition, early endosomes, the major cellular organelles involved in endosomal escape, begin to internalize the nanoparticles 1 h after treatment.^[^ ^8^, ^9^, ^14^, ^35^ ^]^ Therefore, our observation that endosomal escape actively occurred at 40 min after treatment was consistent with previous findings. The slightly earlier occurrence of endosomal escape in our study (by ≈20 min) may be attributed to the differences in the nanoparticle characteristics and cell line used.

Moreover, to demonstrate that this MRI‐based evaluation method can distinguish differrences between endosomal escape efficiency index of various LNP formulations, we conducted in vitro MR experiments using IO@LNP formulated with DOPE (IO@LNP(DOPE)) instead of DSPC, which is known to enhance endosomal escape (Figure 3e,f).^[^ ^12^ ^]^ Although both DSPC and DOPE function as structural helper phospholipids in LNPs, they differ in their ability to promote endosomal escape. DSPC is a saturated lipid that stabilizes the bilayer phase of LNPs but lacks the membrane‐destabilizing properties necessary for efficient endosomal escape. In contrast, DOPE, with its unsaturated hydrocarbon chained tails and fusogenic properties, facilitate enhanced fusion between the LNP membrane with the endosomal membrane.^[^ ^11^, ^35^ ^]^ To specifically isolate the effect of phospholipid composition on endosomal escape, DOTMA, a permanently cationic lipid, was used as the cationic lipid component in all LNP formulations. Notably, IONPs were successfully incorporated into LNPs formulated with both DOTMA and SM‐102—an ionizable lipid utilized in clinically approved LNP systems—while forming uniform particle size and demonstrable MR properties (Figure S10, Supporting Information). Although SM‐102‐based IO@LNP were successfully prepared, the pH‐dependent ionization characteristics of SM‐102 could lead to variations in cellular uptake compared to DOTMA‐based IO@LNP with the pH‐independent cationic charge. Such differences in uptake efficiency would confound the direct comparison of endosomal escape efficiency indices. Thus, the use of DOTMA provided a consistent cationic environment, enabling a more controlled assessment of the impact of phospholipid composition on endosomal escape.

Consequently, DOPE promoted endosomal escape more efficiently than DSPC by destabilizing the endosomal lipid bilayer.^[^ ^12^ ^]^ Compared to the expected maximum *R*_2_ value—calculated by multiplying the actual Fe concentration by *r*_2,loaded_ (21.44) —the overall *R*_2_ values observed in the cells were even lower than those in the previous two cases. This results implied that endosomal escape occured more actively with DOPE. Here, the endosomal escape efficiency index over 40–120 min was 0.41 min^−1^. The highest probability for mRNA escape occurs in early endosomes.^[^ ^8^ ^]^ We therefore hypothesized that increased escape efficiency index during the early stage (0–40 min) of endosomal escape would ultimately result in higher protein expression by facilitating the escape of intact mRNAs. To investigate this, the time‐dependent endosomal escape efficiency index was calculated in the three intervals: 0–40, 40–80, and 80–120 min (Figure 3g). During the early stage (0–40 min), both chloroquine‐treated IO@LNP and IO@LNP(DOPE) showed significantly faster endosomal escape compared to IO@LNP (*p* < 0.05 and *p* < 0.001, respectively) (Figure 3g). These results imply that IO@LNPs remain more entrapped to the endosomes and are less effective at the escape of IONPs compared to IO@LNP(DOPE).^[^ ^12^ ^]^ To further evaluate this, we formulated mRNA‐LNPs with either DOPE (mRNA‐LNP(DOPE)) or DSPC (mRNA‐LNP(DSPC)), and assessed tdTomato protein expression in the transfected cells. As expected, cells treated with mRNA‐LNP(DOPE) exhibited significantly higher mean tdTomato‐fluorescence intensity compared to the DSPC group (*p* < 0.0001) (Figure S11a,b, Supporting Information). The increased endosomal escape of mRNA was due to enhanced fusion with the endosomal membrane.^[^ ^12^ ^]^ Taken together, fusogenic properties of DOPE caused higher MRI‐based endosomal escape efficiency index in the early endosomal stage (0–40 min), and consequently facilitated more efficiency of the mRNA escape. In conclusion, this demonstrated that our measurement technique for escape efficiency index can sensitively distinguish between the endosomal escape efficiencies according to the nanoparticle formulations and experimental conditions used.

At present, most techniques used to assess endosomal escape are based on imaging, which can either track the cargo directly or monitor surrogate markers indicative of escape. For direct visualization, recent improvements in microscopy have enhanced our ability to observe intracellular transport of RNA‐loaded lipid nanoparticles (LNPs) in greater detail. This often involves fluorescent tagging of the payload ^[^ ^26^, ^37^, ^38^, ^39^ ^]^ or employing transmission electron microscopy to visualize gold‐labeled siRNA within LNPs.^[^ ^18^ ^]^ Indirect strategies typically detect markers of endosomal disruption as a sign of escape. Additionally, artificial membrane systems have been applied to study how nanoparticles interact with endosomal membranes.

Earlier studies have shown limitations in analyzing endosomal escape in multicellular or in vivo settings (Table 1). High‐resolution microscopy is commonly used for direct measurement of endosomal escape. This involves calculating the subcellular spatial relationship between the DDSs and endosomes at various stages (Method 1). Methods 2–4 in Table 1 also use microscopy for assessing endosomal escape efficiency. Method 2 utilizes the changes in the DDS fluorescence pattern before and after the escape. However, fluorescent labeling may affect the escape efficiency and, in turn, reduce the accuracy of efficiency estimates. Method 3 utilizes membrane‐impermeable dyes, such as calcein that produces unique fluorescent patterns after escape. Nevertheless, this approach poses a challenge as the efficiency of endosomal escape can vary between small molecules and the DDS. Method 4 assumes a constant signaling protein level throughout the cytoplasm, which may not be true. Notably, Methods 1–4 are not applicable to multicellular or in vivo settings because the content of target materials in the cytosol is usually low.^[^ ^18^ ^]^ However, using fluorescence with sufficient fluorophores may enable multicellular quantification (Methods 5–8). Flow cytometry has been successfully applied for multicellular estimation using these methods.^[^ ^57^, ^58^ ^]^ Nonetheless, these methods have certain limitations. For example, in Method 5, the model cellular membrane does not fully represent the actual endosomal membrane. Method 6 presents two specific challenges. If a DDS is linked to pH‐sensitive moieties, these components should ideally remain consistently bound to the DDS; however, this may not be true. In contrast, if the pH‐sensitive moieties are introduced separately from the DDS, their escape may differ from those of the DDS. Method 7 has a drawback in that surface modification used for Förster resonance energy transfer could affect the endosomal escape efficiency. Method 8, often referred to as a transfection assay, is generally regarded as a standard measure of effectiveness of RNA therapeutics. It directly evaluates the transfected outcomes that are closely tied to therapeutic efficacy. However, owing to the challenges in measuring transfection efficiency and its potential lack of reproducibility, there is a need for LNP quality assessment methods that focus on individual factors closely associated with therapeutic efficacy, such as endosomal escape. Additional steps following RNA escape from endosomes (e.g., RNA transportation to the nucleus, translational efficiency, the duration of protein translation, and degradation of nucleic acids) could complicate the distinction between other factors and LNP effectiveness.^[^ ^35^ ^]^ In addition, variations in the size and chemical composition of nucleic acids may affect the transfection efficiency and hinder the comparison of different studies. For example, the endosomal escape efficiency can be affected by the charge on RNA because RNA molecules carrying a more negative charge experience a stronger mutual repulsive force that facilitates their escape from the LNPs.^[^ ^59^ ^]^ The negative charge on RNA, in turn, is determined by various factors, such as the RNA length, nucleotide sequence, single‐ or double‐stranded structures, and number of attached phosphate groups. Thus, the direct quantification of endosomal escape of DDS is preferable over transfection assays. Notably, Methods 5–8 do not support in vivo imaging. Although techniques such as tomography approaches with fluorescence imaging or advanced microscopy methods such as two‐photon microscopy can capture in vivo images, several challenges persist. These challenges include a limited fluorescence signal intensity, low spatial resolution, poor signal‐to‐noise ratio due to autofluorescence, light scattering, photobleaching, and absorption in deeper tissues. Together, these factors make in vivo imaging practically difficult. In contrast, our approach offers standardization owing to the homogeneity and reproducibility of IONPs and IO@LNPs. Furthermore, the MRI technique enables application for both multicellular and in vivo measurements.

**Table 1:** Comparison of different methods for evaluating the endosomal escape of delivered molecules.

Method	Mechanism	Quantification	Imaging modality	Multicellular evaluation	In vivo applicability	Endosomal escape	References
1	Colocalization and correlation	Correlation with a super‐resolution microscope	Microscope	No	No	1–2% ^[^ ^14^ ^]^ 2–3% ^[^ ^40^ ^]^	[8, 9, 14, 35, 40]
2	Diffusion of escaped material	Fluorescence pattern	Microscope	No	No	NQ	[18, 41, 42, 43]
3	Membrane‐impermeable dye	Super‐resolution microscope	Microscope	No	No	0–42% ^[^ ^48^ ^]^ 0.14–0.3% ^[^ ^49^ ^]^	[44, 45, 46, 47, 48, 49]
4	A signal upon contact with the escaped component and a protein in the cytosol	Super‐resolution microscope	Microscope	No	No	NQ	[50]
5	Membrane damage marker	Fluorescence intensity	Microscope	Yes	Limited	NQ	[22, 51, 52]
6	pH‐sensitive marker in acidic endosomes	ROS intensity	Microscope	Yes	Limited	NQ	[38, 53]
7	Intracellular Dicer reaction, glutathione reaction	FRET‐based fluorescence intensity	Microscope	Yes	Limited	10% ^[^ ^39^ ^]^ < 3% ^[^ ^54^ ^]^	[39, 54, 55, 56]
8	Transfection of a reporter gene (e.g., luciferase)	Fluorescence intensity	Microscope	Yes	Limited	NQ	[18]
This study	IONP dispersal	*R*_2_ change	MRI	Yes	Yes		‐

ROS, reactive oxygen spefies; FRET, Förster resonance energy transfer; IONP, iron oxide nanoparticle; MRI, magnetic resonance imaging; NQ, not quantified

### 2.5 Validation of MRI‐Based Method for Evaluating Endosomal Escape

**Figure 4:**
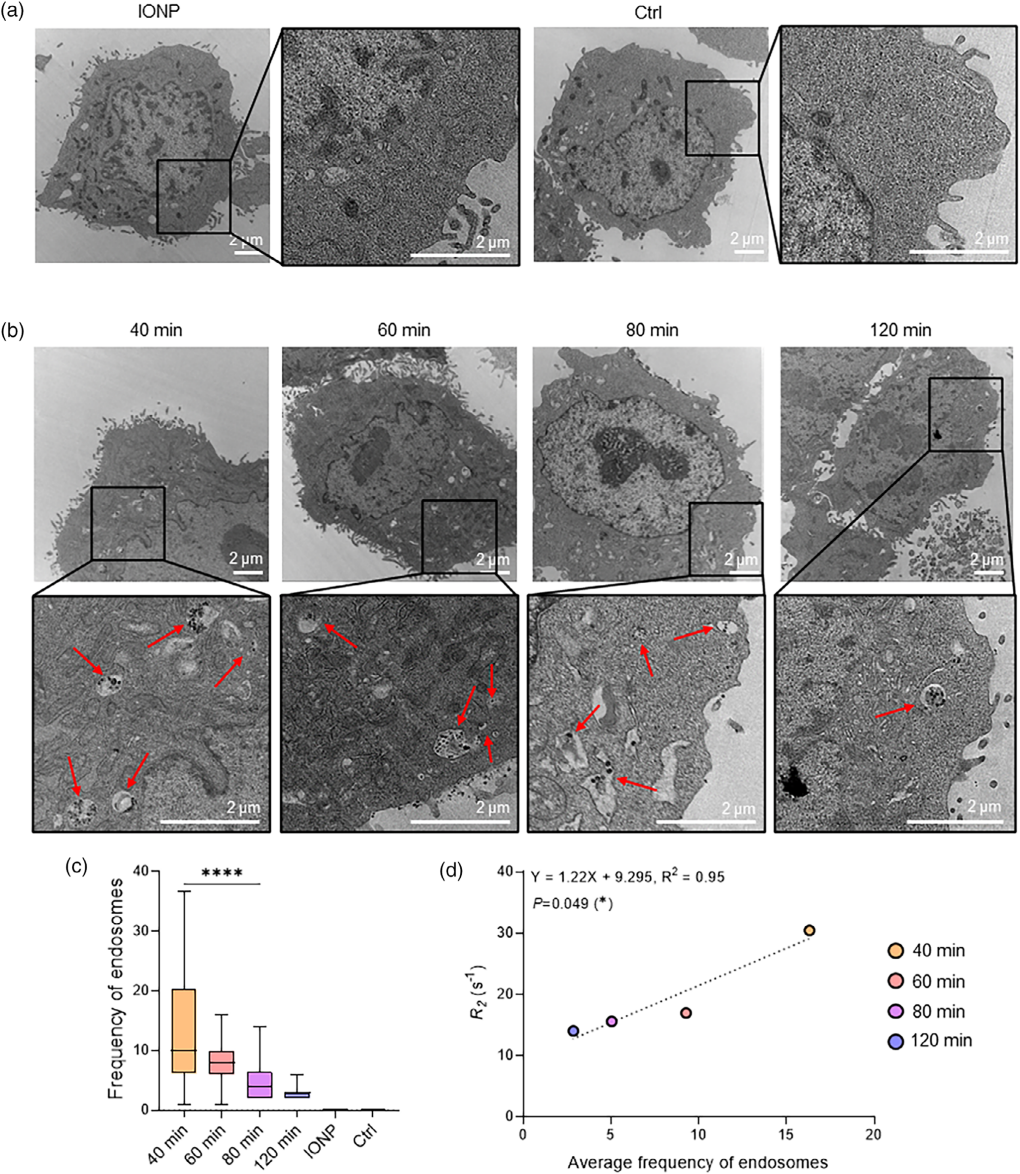
Correlation analysis between Bio‐TEM and in vitro MRI. a,b) Bio‐TEM images of cells incubated with IONPs or IO@LNPs compared with untreated control (n > 30 cells per group). No IONPs or endosome structures are observed in (a), while endosomal structures (red arrows) are clearly visible in IO@LNP‐treated cells in (b). c) Semiquantitative analysis of Bio‐TEM images, displaying the top 50% of cells based on the number of endosomes from at least 30 cells in each group as a quality control process. Only the endosomes containing ≥ 3 IO@LNPs were counted. Following endosomal escape, the number of endosomes decreased. Data presented as Box plot with Min to Max whiskers, *n* = 3, *p*‐values are calculated using ANOVA‐test, ^****^ *p* < 0.0001. d) Correlation analysis between Bio‐TEM quantification and in vitro MRI. With increasing incubation time, a decline in the number of endosomes was observed, in agreement with the decrease in *R*_2_ values. *p*‐values are calculated using F‐test, ^*^ *p* < 0.05.

To validate the in vitro MR results, Bio‐TEM imaging was carried out to observe intracellular vesicles (endosomes) containing IO@LNPs. The DSPC experimental group, which exhibited mild endosomal escape, was selected for validation experiments. No detectable IONP‐containing endosome was found in the IONP‐treated or negative control groups (**Figure** **4**a). However, endosomes with IO@LNP were visible in IO@LNP‐treated cells (Figure 4b). Due to their high structural stability, LNPs are resistant to disassembly within endosomal compartments, thereby limiting the efficient release of encapsulated cargos into the cytosol.^[^ ^60^ ^]^ Instead, cytosolic release of the cargos is facilitated via membrane fusion between the LNP membrane and endosomal membranes, mediated by components such as helper lipids and ionizable lipids. Given the confined volume of the endosomal space, even if IONPs were released and remained trapped within it, their interparticle distances would remain confined to a narrow spatial range. It indicates endosomal entrapment rather than true endosomal escape. Consequently, MRI‐based measurements are expected to selectively reflect endosomal escape efficiency index.

Based on this observation, we calculated the number of endosomal structures containing IO@LNPs in single cells (Figure 4c). The frequency of endosomes containing IO@LNPs decreased as the total incubation time increased. The coefficient of determination (R^2^) between the average frequency of endosomes and *R*_2_ relaxation rate from the in vitro MRI was 0.95 (Figure 4d). Thus, the change in *R*_2_ values observed in the in vitro MRI maps was attributed to endosomal escape.

### 2.6 MRI‐Based In Vivo Quantification of Endosomal Escape

To apply our MRI technique in vivo, we first assessed the delivery of mRNA by LNP in vivo. tdTomato mRNA‐LNP labeled with DiR (mRNA‐LNP/DiR) was synthesized using the same formulation as IO@LNP and administered through intramuscular injection into the hindlimb. DiR fluorescence imaging showed that mRNA‐LNPs stably remain at the injection site (Figure S12a, Supporting information). In addition, we further quantified intra‐tissue level of mRNA using real‐time quantitative polymerase chain reaction (qPCR). The relative tdTomato mRNA level in muscle was significantly higher than in other organs (Figure S12b and Table S1, Supporting information). In contrast, a small amount of tdTomato fluorescent protein was expressed in the muscle tissue (Figure S12c, Supporting information). Taken together, these results indicate that mRNA‐LNPs were successfully delivered to in vivo tissue, and a portion of the delivered mRNAs escaped from endosomes and was translated into proteins. However, most of the delivered mRNA likely failed to endosomally escape, resulting in only a small fraction being translated into protein.

**Figure 5:**
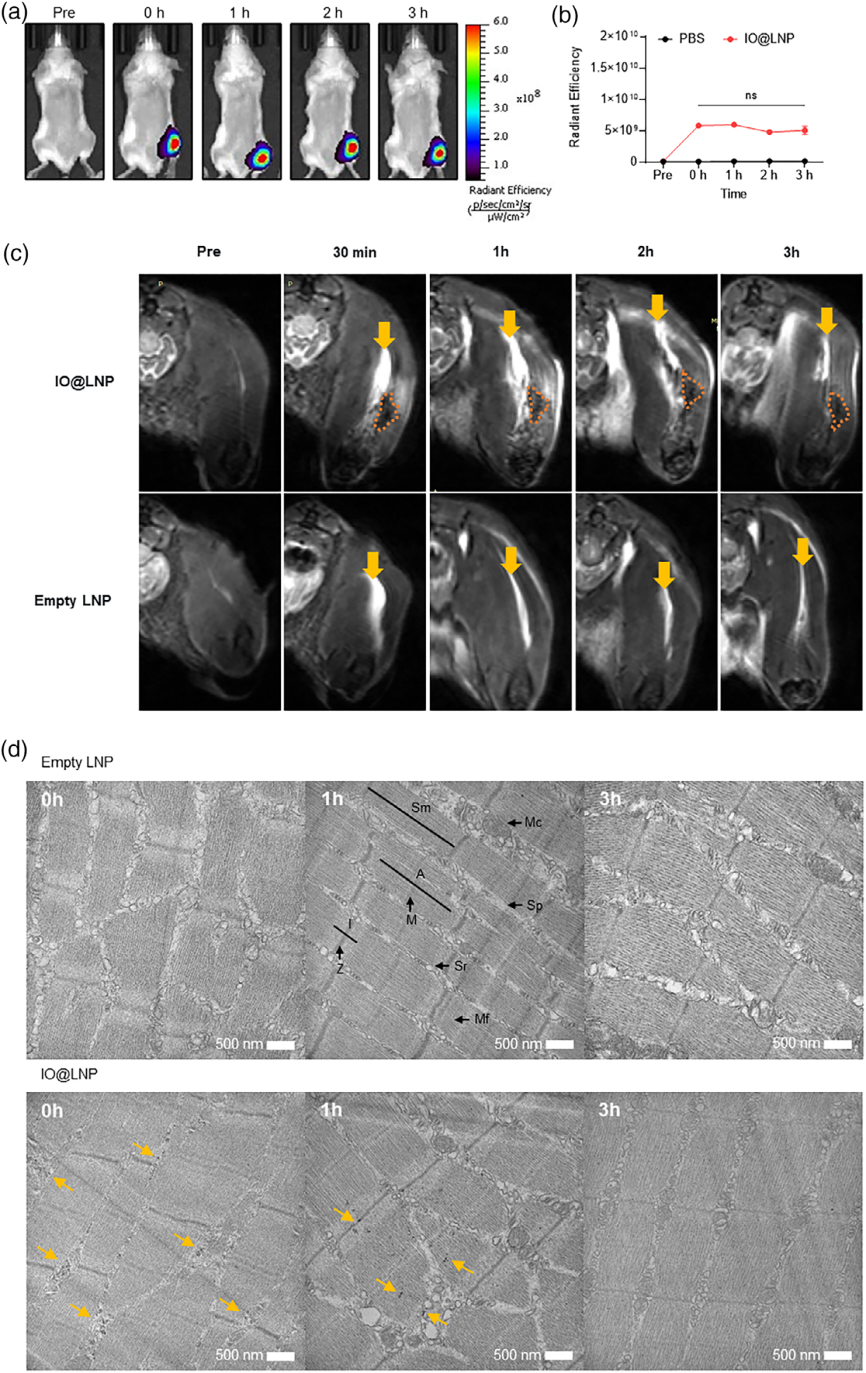
In vivo MRI for evaluating endosomal escape. a) Balb/c mice were each injected with IO@LNP intramuscularly into the right hindlimb. DiR fluorescence images were acquired at 0, 1, 2, and 3 h after injection using an IVIS Spectrum instrument. b) The fluorescence intensity in the injection site was measured over the muscles using an equally sized region of interest, compared with that of PBS‐injected mice. Data presented as mean ± SEM, 3≤n≤14, *p*‐values are calculated using ANOVA‐test. Most of the injection material remained at the injection site. c) *T*_2_‐weighted images before and after the administration of IO@LNPs and empty LNPs. The yellow arrows indicate bright regions that might correspond to the solution, including PBS, whereas the orange dotted lines outline the dark regions that are only specific to IO@LNP. d) Bio‐TEM images according to time and solutions. The histological structure of muscle fibers is clearly visible. The yellow arrows indicate the existence of loaded IONPs within the LNPs. A, A‐band; I, I‐band; M, M‐line; Z, Z‐line; Sm, Sarcomere; Sp, Sarcoplasm; Sr, Sarcoplasmic reticulum; Mc, Mitochondrion; Mf, Myofibril.

To explore the biodistribution of IO@LNP after intramuscular injection, IO@LNPs were labeled with DiR (IO@LNP/DiR) and injected intramuscularly, and fluorescence signals were quantified using an in vivo imaging system (**Figure** **5**a). The radiant efficiency remained stable for 3 h in the muscle but was not observed in other organs (Figure 5b; Figure S13, Supporting Information). This result suggested that the majority of injected LNPs remained localized without diffusing to other organs until at least 3 h after administration. Correspondingly, IO@LNPs and empty LNPs were intramuscularly injected into mouse hindlimbs, and *T*_2_‐weighted images were acquired at different time points (Figure 5c). Following the injection of IO@LNPs, two distinct signals were observed from the muscle tissue: intense bright areas and dark regions. The bright areas, which were also observed in mice injected with empty LNPs, were attributed to the signals most likely caused by the solution of PBS containing IO@LNPs or empty LNPs, as a similar area was observed in mice treated with PBS only. In contrast, the dark regions were exclusive to the IO@LNP‐treated samples, indicating signals associated with IO@LNPs. These dark regions became gradually brighter after administration, implying endosomal escape (Figure S14, Supporting Information).

After treating muscle tissues with phosphate‐buffered saline (PBS), empty LNPs, and IO@LNPs, the tissues were obtained from mice at different time points (0, 1, and 3 h), and Bio‐TEM images were acquired for a validation study (Figure 5d; Figure S15, Supporting Information). Unlike PBS and empty LNPs, treatment with IO@LNPs produced a distinctive pattern caused by the aggregated IONPs (black dots) within the IO@LNPs in the sarcoplasm. Immediately after treatment (i.e., at 0 h), a pattern of IO@LNPs was observed along the spaces between muscle fibers. At 1 h, the IO@LNPs penetrated the muscle fibers, and the nanoparticle pattern was not visible in many fibers. After 3 h, the IO@LNP pattern was difficult to discern. This indicated that IONPs loaded in IO@LNPs were gradually ingested by cells and became dispersed, leading to a significant decrease in the amount of loaded IONPs after 1 h. Therefore, the Bio‐TEM results supported the in vivo MR findings sufficiently.

Efficient RNA delivery by LNPs cannot achieve therapeutic effectiveness unless the RNA molecules successfully escape from the endosome to the cytoplasm, where they perform their intended functions. Endosomal escape represents a significant bottleneck in intracellular delivery, and its efficiency is a critical determinant of the efficacy of RNA‐based therapies. Therefore, LNPs with high endosomal escape efficiency are considered promising drug vehicles for RNA delivery.^[^ ^8^ ^]^ In this study, we proposed the use of MRI to measure the efficiency index of LNP‐mediated endosomal escape in vivo. Ultrasmall, uncoated IONPs with negative charges were employed to leverage the advantages of the electrostatically mediated encapsulation within the LNPs. Our results revealed a substantial decrease in the *r*_2_ value of IONPs following their escape from LNPs and endosomes. The in vitro endosomal escape efficiency index of IO@LNPs was successfully measured, and the potential of the method for in vivo measurements was demonstrated.

Most DDS studies on mRNA delivery assess the extent of translation from mRNA to protein using luciferase mRNA.^[^ ^61^, ^62^, ^63^ ^]^ However, our method, which employs IONPs with reproducible sizes and charges, may act as an optimal surrogate for evaluating endosomal escape. Notably, our results obtained in the presence of chloroquine, a chemical known to enhance endosomal escape, suggested that the change in the *R*_2_ signal was largely dependent on endosomal escape. Our MRI‐based quantification method can be applied to cases where mRNA transfection and protein translation are challenging, such as with the 4T1 cell line. Furthermore, our technology can differentiate between various LNP formulations, indicating that it can play a critical role in comparing endosomal escape efficiency indices across different LNP formulations in RNA‐LNP research and screening diverse DDS types aimed at nucleic acid delivery.

Despite our promising results, the proposed method had certain limitations. First, although this study considered only two LNP compositions (DSPC vs DOPE), it is necessary to compare the results obtained using various compositions to check if the endosomal escape efficiency index is truly linked to therapeutic efficacy. Similarly, the number of cell lines used should be expanded. Second, the estimated endosomal escape efficiency index of ≈1 min^−1^ is significantly higher than what has been reported in the literature and our preliminary results using Tomocube. This discrepancy likely arises because MR‐based quantification serves as an indirect measure and does not replicate RNA‐LNP conditions exactly. For instance, the steric hindrance of bulky IONPs within LNPs can weaken the encapsulation capability of LNPs and facilitate endosomal escape efficiency of them. Nevertheless, we believe that this distinction primarily enhances the signal difference, enabling a faster evaluation of LNPs. In fact, we previously attempted to prepare LNPs loaded with both IONPs and mRNA (IONP‐mRNA‐LNPs), but while ≈90% of the mRNA was successfully loaded, the IONPs were poorly incorporated. As a result, using this system could lead to a reduced *R*_2_ signal, raising concerns about the accuracy of the endosomal escape efficiency measurement. Finally, as this study relies on ultrasmall, unmodified IONPs, examining the effect of the exocytosis,^[^ ^64^ ^]^ and biodegradation ^[^ ^65^ ^]^ of IONPs is essential. Some cells released several extracellular vesicles in the Bio‐TEM images at 120 min (Figure 4b). This implied that the decrease in the number of endosomes, particularly at later time points, as shown in Figure 3f, may be affected by active exocytosis.^[^ ^64^ ^]^ To address this, we proposed a method to measure the decomposition or exocytosis ratio (η) by utilizing both *R*_1_ and *R*_2_ maps when the iron concentration was already experimentally measured (Supporting Information).

## 3 Conclusion

There is no doubt endosomal escape by LNPs is a critical barrier to maximising the potential of therapeutics such as nucleic acid and is still poorly understood and quantified. In this study, we successfully developed an approach for measuring the endosomal escape efficiency index by LNPs using MRI. To achieve this, we used ultrasmall iron oxide‐loaded LNPs and compared between the *R*_1_ and *R*_2_ MRI parameters of these particles and their diffused state within cells. This approach not only identified the potential of reproducibly synthesized ultrasmall iron oxide as an optimal reference for endosomal escape efficiency but also introduced a method for the in vivo measurement of endosomal escape. This breakthrough opens new possibilities for understanding the intercellular heterogeneity of endosomal escape within biological systems.

## 4 Experimental Section

### Materials

Anhydrous ethanol (C_2_H_6_O), anhydrous sodium acetate (CH_3_COONa), acetic acid (CH_3_COOH), ferric chloride hexahydrate (FeCl_3_∙6H_2_O), trisodium citrate dihydrate (Na_3_Cit∙2H_2_O), diethylene glycol, DSPC, DOTMA, cholesterol, 1,1′‐dioctadecyl‐3,3,3′,3′‐tetramethylindodicarbocyanine 4‐chlorobenzenesulfonate salt (DiD), Triton X‐100, Hoechst 33342 solution, and low melting agarose were purchased from Sigma‐Aldrich (St. Louis, MO, USA). 1,2‐distearoyl‐sn‐glycero‐3‐phosphoethanolamine‐N‐[methoxy(polyethylene glycol)‐5000] (mPEG‐DSPE) was purchased from Creative PEGWorks (Durham, NC, USA). 1,1′‐dioctadecyl‐3,3,3′,3′‐tetramethylindotricarbocyanine iodide (DiR) was purchased from MedChemExpress (Monmouth Junction, NJ, USA). PBS was purchased from BYLABS (CA, USA). The plasmid containing condon‐optimized tdTomato DNA was purchased from BIONICS (Seoul, South Korea). Polymerase chain reaction (PCR) purification kit was purchased from Bioneer (Daejeon, South Korea). T7 RNA polymerase, DNase I, and Antarctic phosphatase were purchased from Enzynomics (Daejeon, South Korea). Tris‐borate‐EDTA buffer and Quant‐it RiboGreen RNA Assay Kit, and Lipofectamine MessengerMAX Reagent were purchased from Thermo Fisher Scientific (Waltham, MA, USA). Roswell Park Memorial Institute (RPMI) 1640 medium was purchased from WELGENE (Gyeongsan, South Korea). DPBS and fetal bovine serum (FBS) were purchased from Cytiva (Logan, UT, USA). Antibiotics‐antimycotics and 0.5% Trypsin‐EDTA were purchased from Gibco (Grand Island, NY, USA). 4% paraformaldehyde was purchased from Biosesang (Seongnam, South Korea). Reagents for Karnovsky's Fixative for Bio‐TEM samples, including sodium cacodylate buffer, glutaraldehyde, uranyl acetate, and Spurr's Resin, were provided by National Instrumentation Center for Environmental Management (NICEM, South Korea). QIAzol Lysis Reagent was purchased from QIAGEN (NRW, Germany). PrimeScript RT Master Mix was purchased from TAKARA BIO Inc. (Shiga, Japan).

### Solvothermal Synthesis of IONPs

Citrate‐stabilized ultrasmall IONPs were synthesized using the solvothermal method in accordance with a previous study.^[^ ^18^ ^]^ First, FeCl_3_·6H_2_O (270.25 mg, 1.00 mmol) was dissolved in diethylene glycol (10 mL) under stirring at 100 × g. This solution was mixed with Na_3_Cit·2H_2_O (117.75 mg and 0.40 mmol) and heated at 80°C for 2 h. Anhydrous sodium acetate (328 mg and 4.00 mmol) was then added, and the mixture was heated at 200°C for 4 h. After cooling to 100°C, ethanol was added to the reaction mixture and centrifuged at 9000 × g for 30 min. The supernatant was discarded. The iron oxide sediment was washed with ethanol, separated by centrifugation at 6000 × g for 30 min, and dried overnight at 25°C (room temperature).

### Self‐Assembly of IO@LNPs

The chemical composition of the LNPs was based on a previous study.^[^ ^66^ ^]^ Cationic (e.g., DOTMA) and ionizable lipids can encapsulate negatively charged molecules, including nucleic acids. Cationic lipids were used in this study to facilitate the reproducible comparision with other conditions. The mixed lipids (DOTMA/DSPC/cholesterol/PEG or DOTMA/DOPE/cholesterol/PEG = 50/10/38.5/1.5 mol%) were dissolved in ethanol to achieve a final total lipid concentration of 10.4 mg mL^−1^. The lipids were mixed with 25 mm sodium acetate buffer (pH 4.0) containing the IONPs (Fe: lipid = 1/20 w/w) using a T‐junction system and a Gemini 88 Plus Dual Rate syringe pump (KD Scientific Inc., USA) at a constant flow rate. This ethanol–water formulation was then enclosed in Spectra/Por 2 dialysis membrane (Molecular weight cut‐off: 12–14 kD; Spectrum Labs, USA) and dialyzed in 1000‐fold volume of 1 × PBS overnight to remove ethanol.

A lipophilic fluorescent dye (DiD) was applied to IO@LNPs to allow visualization of the LNPs under a fluorescence microscope. IO@LNP/DiD was prepared following the same procedure as IO@LNPs, except for the addition of 4 µL of DiD into the lipid‐ethanol phase.

### Physicochemical Characterization of IONPs and IO@LNPs

The FTIR spectrum of IONPs was obtained using the Platinum attenuated total reflection FTIR spectrometer (Bruker, Germany). A VSM test was performed to identify the superparamagnetic properties of IONPs using VSM‐7410 (Lake Shore, USA). The size of IONPs was confirmed using the LIBRA 120 energy‐filtering transmission electron microscope (Carl Zeiss, Germany). TEM imaging for empty LNPs and IO@LNPs was conducted using Talos L120S cryo‐EM (FEI, Czech Republic) to visually confirm that IONPs were loaded in IO@LNPs. In addition, the size distribution, PDI, and zeta potentials of nanoparticles (IONPs, IO@LNPs, and empty LNPs) were measured on a Zetasizer Nano ZS (Malvern, UK) or Litesizer 500 (Anton paar, Austria). The Fe concentration per milliliter of LNP was measured using Inductively Coupled Plasma Atomic Emission Spectroscopy (ICP5800, ICP‐AES) (Agilent Technologies, USA).

To further confirm the surface charge and encapsulation of IONPs into LNPs according to the zeta potential results, electrophoresis was performed using an electrophoresis device (Allsheng, China). The samples containing IONPs (8 µg in each well) were loaded onto a 1% agarose gel. Subsequently, electrophoresis was performed using 1X Tris‐borate‐EDTA buffer at 100 V, and a gel image was obtained.

To indirectly evaluate the stability of the particles, IONPs or IO@LNPs were incubated with PBS or acetate buffer. The size based on DLS was monitored according to time using the Zetasizer Nano ZS or Litesizer 500.

### IVT mRNA Synthesis

The DNA sequence encoding the tdTomato protein was codon‐optimized for *Mus musculus*. Then, to facilitate real‐time qPCR analysis, codon scrambling was performed to eliminate tandem repeat DNA sequences of tdTomato at the nucleotide level, while preserving the tandemly repeated amino acid sequence. The plasmid contained the tdTomato open reading frame flanked by 5′ and 3′ untranslated regions. The plasmid DNA was linearized by Spe I digestion at 37°C. Poly‐T tail PCR of linear DNA was performed for tailing poly(A) of mRNA. The PCR product was purified using a PCR purification kit and subsequently used as a template for T7 RNA polymerase‐mediated transcription, according to the manufacturer's instructions. After transcription, the template DNA was digested using DNase I at 37°C for 15 min. Anti‐reverse cap analog (ARCA)‐capped mRNA was purified using an RNA precipitation method and dephosphorylated using the Antarctic phosphatase at 37°C for 30 min. Finally, the synthesized tdTomato mRNA was purified and its integrity was evaluated via 1X Tris‐acetate‐EDTA agarose gel electrophoresis. The capping efficiency was validated via confocal laser scanning microscopy after mRNA transfection into HEK293FT cells using the Lipofectamine MessengerMAX Reagent. The highly concentrated mRNA was kept frozen at −20°C until use.

### Production and Transfection of mRNA‐LNPs

For LNP encapsulation, mRNA was diluted in sodium acetate (pH 4.0) and naturally loaded through self‐assembly production. The concentration of the LNP‐encapsulated mRNA was quantified using the Quant‐it™ RiboGreen RNA Assay Kit. Flow cytometry was performed to evaluate the expression of the tdTomato fluorescent protein. First, HEK293FT cells were incubated with mRNA‐LNPs at 37°C in a humidified 5% CO_2_ incubator for 24 h. After incubation, cells were washed with DPBS twice and suspended in DPBS. Cells were analyzed using FACSymphony A5 instrument (BD Biosciences, USA). tdTomato is an orange fluorescent protein with an excitation peak at 554 nm and emission peak at 581 nm; its detection was performed using a phycoerythrin laser under flow cytometry.

### In‐Solution MRI Phantom of IO@LNPs

To compare the MR characteristics of IONPs, IO@LNPs, and empty LNPs, MRI phantom experiments were performed using a Powerscan MRS 7024 instrument (MR Solutions, UK). An MRI scanner with a high magnetic field of 7.0 T was used to improve the resolution and contrast. The samples were prepared by serial dilution with saline (concentration levels: 1/1, 1/2, 1/3, 1/4, and 1/5) and placed in 0.2 mL PCR tubes. The IONP, IO@LNP, and empty LNP samples at the concentration of 1/1 contained 0.82 955 mg mL^−1^ Fe and 0 mg mL^−1^ lipid, 0.23 065 mg mL^−1^ Fe and 4.33 mg mL^−1^ lipid, and 0 mg mL^−1^ Fe and 4.33 mg mL^−1^ lipid, respectively. The iron concentration was quantified using inductively coupled plasma atomic emission spectroscopy. The MRI sequence parameters for the *R*_1_ and *R*_2_ relaxation rates are presented in Table S2 (Supporting Information).

### In‐Solution MRI Phantom of Dissolved IO@LNPs

Changes in the MR signal according to the dispersion of adjacent IONPs within the LNPs were evaluated by dissolving the IO@LNPs using a detergent. The IO@LNP solution was mixed with 2% Triton X‐100, incubated at 37°C for 10 min, and cooled at 25°C for 5 min. Samples were prepared at concentration levels ranging from 1/1 to 1/5. The IO@LNP samples treated with Triton X‐100 at the concentration level of 1/1 contained 0.13 786 mg mL^−1^ Fe and 2.59 mg mL^−1^ lipid. Finally, the MR signal was measured using a 7.0 T MRI scanner, with the MRI sequence parameters presented in Tables S3 and S4 (Supporting Information).

### Cell Culture and Confocal Imaging

4T1 cells were maintained at 37°C in a humidified 5% CO_2_ incubator in RPMI medium containing 10% FBS and 1% antibiotics‐antimycotics. Cells were seeded in a confocal dish (SPL Life Sciences, South Korea), at a seeding density of 7 × 10^4^ cells per dish, using an EVE automatic cell counter (NanoEnTek, South Korea) and incubated overnight. Cells were treated with IO@LNP/DiD on the dish and then incubated for different times (0, 20, 40, 60, and 80 min) at 37°C. Hoechst 33 342 fluorescent dye (0.5 µL per dish) was used for staining the nuclei. Cells were washed with DPBS to remove the remaining IO@LNPs that were not absorbed by the cells and observed under an A1R confocal laser scanning microscope system (Nikon instruments, Japan). The fluorescence intensity profiles of the cross‐sectional scanned cells (*n* = 15) were analyzed using NIS‐Elements Advanced Research software (Nikon Instruments, Japan).

### In Vitro MRI

The protocol for in vitro MRI has been summarized in the literature.^[^ ^67^, ^68^ ^]^ Briefly, 4T1 cells were cultured in T‐flasks, washed with DPBS, and initially incubated for 40 min in fresh RPMI medium containing IO@LNP/DiD. After changing the medium, the samples were further incubated for 0, 20, 40, and 80 min. Then, cells were washed with DPBS and detached from the flasks after 1 × trypsin‐EDTA treatment. Cell pellets obtained by centrifugation were resuspended in 1 mL DPBS, and the total cell density and viability for each sample were calculated using an automatic cell counter. Cells (ranging from 1.7 × 10^7^ to 2.2 × 10^7^ cells mL^−1^ for each experimental group) were transferred to a microtube, and cell pellets were collected by centrifugation for 10 min at 500 × g and 4°C. The pellets were then resuspended with 4% paraformaldehyde (PFA), incubated for 15 min at 25°C, centrifuged again for 10 min at 500 × g, and rinsed with DPBS. Cells were further centrifuged, resuspended in DPBS (50 µL), and transferred over 4% agarose‐PBS gel in PCR tubes. Agarose gel was used to equal the height of the cell pellets. After the complete precipitation of cells, the supernatant was removed. The resultant samples were stored at 4°C. The *R*_1_ and *R*_2_ values were acquired as described in Table S5 (Supporting Information). During analysis of the MRI map, five different concentrations were employed to create a regression curve linking the Fe concentration with the MR relaxation rate. The MRI experiments were repeated three times for each condition.

### Biological TEM (Bio‐TEM) Imaging

Samples of 4T1 cells treated with IONPs or IO@LNPs were prepared using the same method as that for in vitro MRI until cell fixation. The cell pellets collected in each microtube were treated with Karnovsky's Fixative to preserve the biological samples for electron microscopy. During primary fixation, cells were incubated overnight at 4°C in 0.05 m sodium cacodylate buffer (pH 7.4), 2% PFA, and 2% glutaraldehyde. Subsequently, cells were washed with 0.05 m sodium cacodylate buffer, 1% osmium tetroxide diluted with 0.1 m sodium cacodylate buffer was added, cells were further incubated for 2 h at 4°C, washed with distilled water, and then incubated in 0.5% uranyl acetate overnight at 4°C for En Bloc staining. Subsequently, the samples were washed with deionized water and gradually dehydrated using an ethanol gradient (30%, 50%, 70%, 80%, 90%, and 100%). Spurr's resin was applied for transition and embedding, and then, the samples were incubated overnight at 70°C for polymerization. The solidified samples were sectioned into thin films using an EM UC7 ultramicrotome (Leica, Germany). Finally, Bio‐TEM images were acquired to observe the intracellular IO@LNPs.

For semiquantitative analysis of the Bio‐TEM results, two criteria are established for identifying IO@LNPs within intracellular vesicles (endosomes). First, the signal should be significantly lower than that in the surrounding cytoplasm. Second, the vesicle should include at least three black dots (IO@LNPs). Only distinct cells were considered for semiquantitative analysis among the selected endosome‐containing cells. The number of endosomes in each of these cells was counted and adjusted based on the exposed surface area relative to the total surface area of each cell. In addition, only the top 50% of cells according to the number of endosomes were considered from at least 30 cells in each group as a quality control process. Subsequently, a regression curve was generated between the time points and frequency of endosomes. The frequency of endosomes per cell was plotted against the *R*_2_ value at the corresponding time point.

### In Vivo Experiments

All animal experiments were performed in accordance with the guidelines of the Seoul National University Bundang Hospital (approval number: BA‐2102‐314‐012‐05). Balb/c mice were purchased from OrientBio Inc. All mice undergoing the experiments anesthetized using isoflurane via a gas anesthesia machine. Female BALB/c mice (6 weeks old) were injected with tdTomato mRNA‐LNP/DiR (0.5 mg kg^−1^) or IO@LNP/DiR (1.81 mg kg^−1^) intramuscularly in their hindlimbs. The harvested organs were homogenized, and the RNA was extracted from the tissue lysates using QIAzol Lysis Reagent, according to the manufacturer's instructions. Reverse‐transcriptase PCR (RT‐PCR) was performed to synthesize cDNA from total RNA containing tdTomato mRNA. qPCR was then conducted to quantify the level of tdTomato mRNA delivered to each tissue. The in vivo distribution of IO@LNPs after a pre‐injection and at 0, 1, 2, and 3 h after injection was investigated using IVIS. To acquire the MR signal of IO@LNP and empty LNP in vivo, BALB/c mice were properly fixed in a mold and subjected to breath gating before acquiring the *T*_2_‐weighted images (pre‐injection) using a 7.0 T MRI scanner. Then, *T*_2_‐weighted images were longitudinally acquired at the following time points after injection: 0, 1, 2, and 3 h. In addition, the MR sequence for *T*_2_‐weighted images was FSE26 (batch size: 0; echo spacing: 15; gating mode: 2; averages: 4; no discards: 0; samples: 256; 3D slab thk: 80%; TE: 45 ms; TR: 3000 ms; PE order: 0; echo train: 7; fat saturation: Yes; gate after TR: No; navigator: Yes; post ETL crusher: No). The images were quantitatively evaluated against the neighboring tissue in which the MR contrast effect was not observed.

The number of tissue slices was set to 13, and the median slice was selected for subsequent quantitative measurements. The muscle tissue was treated with PBS, empty LNP, and IO@LNPs using the same administration route, and after 0, 1, and 3 h, the same Bio‐TEM process was applied, including Karnovsky's Fixation.

### Statistical Analysis

Statistical analyses and graph preparation were conducted using GraphPad Prism software 8.4.2 and the *aov* function in *R*. One‐way ANOVA‐test or T‐test was carried out to evaluate the statistical significance of variations between groups. Also, regression analysis was carried out to identify statistical significance of temporal behaviors of nanoparticles using regression analysis, where F‐test statistic was used for computing *p*‐values. The presentation of data were expressed as the mean ± SEM with three or more repeated independent experiments. The specific details of the applied statistical tests are described in each figure legend. In all cases, significance was defined as *p* ≤ 0.05. Statistical significant value was presented as: ^*^ *p* < 0.05, ^**^ *p* < 0.01, ^***^ *p* < 0.001, and ^****^ *p* < 0.0001.

## Conflict of Interest

Hyung‐Jun Im is a co‐founder and Chief Scientific Officer of Portrai.

## Supporting information



Supporting Information

## Data Availability

The data that support the findings of this study are available from the corresponding author upon reasonable request.
