# Supplementary material for: Magnetic Resonance Imaging‐Based Quantification of Endosomal Escape Using Iron Oxide Nanoparticle‐Loaded Lipid Nanoparticles
Source: Adv Healthc Mater. 2025 Aug 5;14(30):e03055. doi: 10.1002/adhm.202503055 (PMC12645086; doi:10.1002/adhm.202503055)
Supplement: Supplementary file 1 — Supporting Information [file ADHM-14-0-s001.docx]

Supporting Information

**Magnetic Resonance Imaging-based Quantification of Endosomal Escape Using Iron Oxide Nanoparticle-loaded Lipid Nanoparticles**

*Somin Lee, Jeongbin Park, Han Na Jung, Shengjun Li, Zhijun Lin, and Hyung-Jun Im**

**Interpretation of MR Signal**

Determination of *R*_1_ and *R*_2_ values and acquisition of parametric images were performed using the pMRI software ([www.parametricmri.com](http://www.parametricmri.com)). In the inversion recovery spin echo (IRSE) sequence, the longitudinal magnetization intensity $I_{z}$ was determined using Equation **(1)**:^[1]^

Equation **(1)**

$$I_{z}=M_{z}^{0}\left[ 1-2\exp\left\{ -\frac{\mathrm{TI}}{T_{1}} \right\}+\exp\left\{ -\frac{\mathrm{TR}}{T_{1}} \right\} \right],$$

$$R_{1}=R_{1,0}+r_{1}C=\frac{1}{T_{1}}$$

where $R_{1}$ is *T*_1_ relaxation rate (s^-1^), $R_{1,0}$ is *T*_1_ relaxation rate (s^-1^) in the absence of IONPs, $r_{1}$ is *T*_1_ relaxation rate per Fe concentration (s^-1^ [mg/mL]^-1^), $C$ is the Fe concentration (mg/mL), $\mathrm{TI}$ is inversion time (s), and $\mathrm{TR}$ is repetition time (s). In inversion recovery fast low angle shot (IR FLASH), *R*_1_ can be illustrated in a similar way to IRSE; however, in this study, we also considered the flip angle, which was eight degrees.

In multi-echo spin-echo sequence (MEMS), the transverse magnetization intensity $I_{xy}$ was determined using Equation **(2)**:

Equation **(2)**

$$I_{xy}=M_{xy}^{0}\exp\left\{ -\frac{\mathrm{TE}}{T_{2}} \right\},$$

$$R_{2}=R_{2,0}+r_{2}C=\frac{1}{T_{2}}$$

where *R*_2_ is the *T*_2_ relaxation rate (s^-1^), *R*_2,0_ is the *T*_2_ relaxation rate (s^-1^) in the absence of IONPs, $r_{2}$ is the *T*_2_ relaxation rate per Fe concentration (s^-1^ [mg/mL]^-1^), $C$ is the Fe concentration (mg/mL), and TE is the echo time (s). In addition, the α value was defined as Equation **(3)**:

Equation **(3)**

$$\alpha=\frac{r_{2}}{r_{1}}$$

Generally, if the α value is less than 5, the contrast agent is classified as a *T*_1_ agent; otherwise, it is classified as a *T*_2_ agent.^[2]^

As the size of magnetic materials increases, their magnetic moment also increases.^[3]^ This can be indicated by a bigger *R*_2_ value, which increases proportionally to the squared magnetic moment, as Equation **(4)**:

Equation **(4)**

$$R_{2}=\frac{\alpha}{d_{\mathrm{NP}}D}\gamma^{2}\mu^{2}C_{\mathrm{NP}}J\left( \omega, \tau_{D} \right)$$

where $d_{\mathrm{NP}}$ is the diameter of IONPs, $D$ is the diffusion coefficient, $\mu$ is the magnetic moment of IONPs, $\gamma$ is the gyromagnetic ratio of water protons, $C_{\mathrm{NP}}$ is the concentration of IONPs, and $J(\omega, \tau_{D})$ is the spectral density function. Clustered magnetic particles show higher *R*_2_ values, whereas monodispersed particles show smaller *R*_2_ values, even if the total iron concentration is the same.^[4]^

When the proportion of loaded IONPs among the total IONPs is defined as $\theta$, the index of the endosomal escape $\frac{\partial\theta}{\partial t}$ can be calculated using the chain rule as Equation **(5)**:

Equation **(5)**

$$\frac{dr_{2}}{dt}=\frac{\partial r_{2}}{\partial\theta} \times\frac{\partial\theta}{\partial t}$$

In this study, the *r*_2_ value of loaded IONPs did not considerably depend on the Fe-to-lipid ratio (w/w). This implies Equation **(6)**:

Equation **(6)**

$$\frac{\partial^{2}r_{2}}{\partial\theta^{2}}=0 \Leftrightarrow\frac{\partial r_{2}}{\partial\theta}=r_{2, \mathrm{loaded}}-r_{2, \mathrm{free}}$$

where $r_{2}$ is the *T*_2_ relaxation rate per iron concentration and $\theta$ is the proportion of loaded IONPs among the total IONPs.

Theoretically, the decomposition or exocytosis ratio ($\eta$) and index of endosomal escape can be simultaneously estimated, using ultrasmall IONPs that can be used as *T*_1_ and *T*_2_ dual-mode contrast agents as Equation **(7)**.

$$r_{1}\left( t \right)=\theta r_{1, \mathrm{loaded}}+\left( 1-\eta\right)\left( 1- \theta\right)r_{1, \mathrm{free}}\begin{matrix} \\ \\ \end{matrix}$$

$$r_{2}\left( t \right)= \theta r_{2, \mathrm{loaded}}+\left( 1-\eta\right)\left( 1- \theta\right)r_{2, \mathrm{free}}\begin{matrix} \\ \\ \end{matrix}$$

$$\therefore\left( \begin{matrix} \frac{dr_{1}\left( t \right)}{dt} \\ \frac{dr_{2}\left( t \right)}{dt} \end{matrix} \right)= \left( \begin{matrix} r_{1, \mathrm{loaded}} & r_{1, \mathrm{free}} \\ r_{2, \mathrm{loaded}} & r_{2, \mathrm{free}} \end{matrix} \right)\left( \begin{matrix} \frac{d\theta}{dt} \\ -\left( 1-\eta\right)\frac{d\theta}{dt} \end{matrix} \right)$$

Equation **(7)**

**Calculation of Endosomal Escape Efficiency Index**

The endosomal escape index $\frac{\partial\theta}{\partial t}$ was calculated using Equation **(8)**:

Equation **(8)**

$$\frac{\partial\theta}{\partial t}= \frac{1}{C\left( r_{2, \mathrm{loaded}}-r_{2, \mathrm{free}} \right)}\frac{dR_{2}}{dt},$$

where $\theta$ is the proportion of loaded IONPs among total IONPs, $\frac{dR_{2}}{dt}$ is the decrease in $R_{2}$ values over time, $r_{2, \mathrm{loaded}}$ (the *r*_2_ value for IO@LNPs) is 21.44 s^-1^[mg/mL Fe]^-1^, $r_{2, \mathrm{free}}$ (the *r*_2_ value for dispersed IONPs that have escaped from LNPs) is 2.83 s^-1^[mg/mL Fe]^-1^, and $C$ is the Fe concentration of the MR plane.

Quantifying the iron concentration for in vitro MR experiments is challenging because estimating the slice volume is difficult. However, quantifying the Fe concentration using *r*_1_ and *r*_2_ values is possible. If the concentrations of IO@LNP and IONP at an arbitrary time point $t$ are $C_{IO@LNP, t}$ and $C_{\mathrm{IONP}, t}$, respectively, Equation **(9)** can be obtained.

Equation **(9)**

$$R_{1, t}=r_{1, \mathrm{loaded}} C_{IO@LNP, t}+ r_{1, \mathrm{free}} C_{\mathrm{IONP}, t}+R_{1, cell only}$$

$$R_{2, t}=r_{2, \mathrm{loaded}} C_{IO@LNP, t}+ r_{2, \mathrm{free}} C_{\mathrm{IONP}, t}+R_{2, cell only}$$

$$\therefore\left( \begin{matrix} C_{IO@LNP, t} \\ C_{\mathrm{IONP}, t} \end{matrix} \right)= \left( \begin{matrix} r_{1, \mathrm{loaded}} & r_{1, \mathrm{free}} \\ r_{2, \mathrm{loaded}} & r_{2, \mathrm{free}} \end{matrix} \right)^{-1}\left( \begin{matrix} R_{1, t}-R_{1, cell only} \\ R_{2,t}-R_{2, cell only} \end{matrix} \right)$$

Thus, given $t$ is 40 min, $r_{1, \mathrm{loaded}}$ is 0.061 s^-1^[mg/mL Fe]^-1^, $r_{1, \mathrm{free}}$ is 0.35 s^-1^[mg/mL Fe]^-1^, $r_{2, \mathrm{loaded}}$ is 21.44 s^-1^[mg/mL Fe]^-1^, $r_{2, \mathrm{free}}$ is 2.83 s^-1^[mg/mL Fe]^-1^, $R_{1, t}$ is 0.54 s^-1^, $R_{1, cell only}$ is 0.47 s^-1^, $R_{2, t}$ is 30.65 s^-1^, and $R_{2, cell only}$ is 6.40 s^-1^, the iron concentration $C$, which is the sum of $C_{IO@LNP}$ and $C_{\mathrm{IONP}}$, was 1.13 mg/mL, and was mostly composed of IO@LNP at 40 min.

We verified this calculation by measuring the iron concentration using inductively coupled plasma-atomic emission spectroscopy. Consequently, the Fe-to-cell ratio for the resuspended 40-min sample was 1.96 × 10^-7^ µg/cells. Therefore, assuming without empty space between cells, the effective volume of cells was 173.45 μm^3^ as Equation **(10)**.

Equation **(10)**

$$1.13 \frac{\mathrm{mg}}{\mathrm{mL}} \mathrm{Fe}\simeq1.96 \times{10}^{-7} \frac{\mu g}{\mathrm{cells}} \div effective volume$$

This value was slightly larger than that of a red blood cell (~100 µm^3^), supporting our experimental condition for in vitro MR, in which 4T1 cells were densely compressed. Furthermore, the Fe-to-cell ratio did not demonstrate significant variation over time (standard deviation = 3.56 × 10^-8^ µg/cells), or substantial differences compared with that of DOPE-IO@LNP (difference = 7.08 × 10^-9^ µg/cells). This indicated that the iron uptake of cells remained considerably constant in this study.


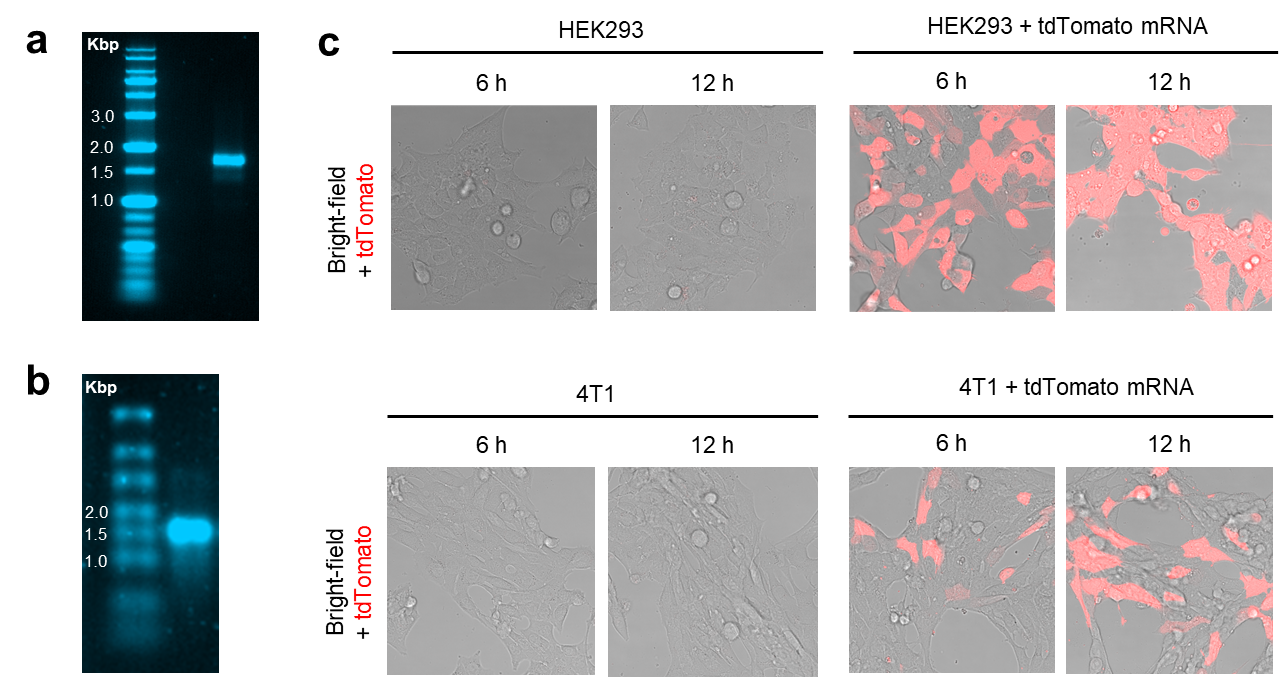


**Figure S1. In vitro transcription and fluorescent protein expression of tdTomato mRNA.** (a, b) Agarose gel electrophoresis images of tdTomato DNA tailed with poly-T (a) and tdTomato mRNA (b). (c) Fluorescence image of cells transfected with capped tdTomato mRNA.


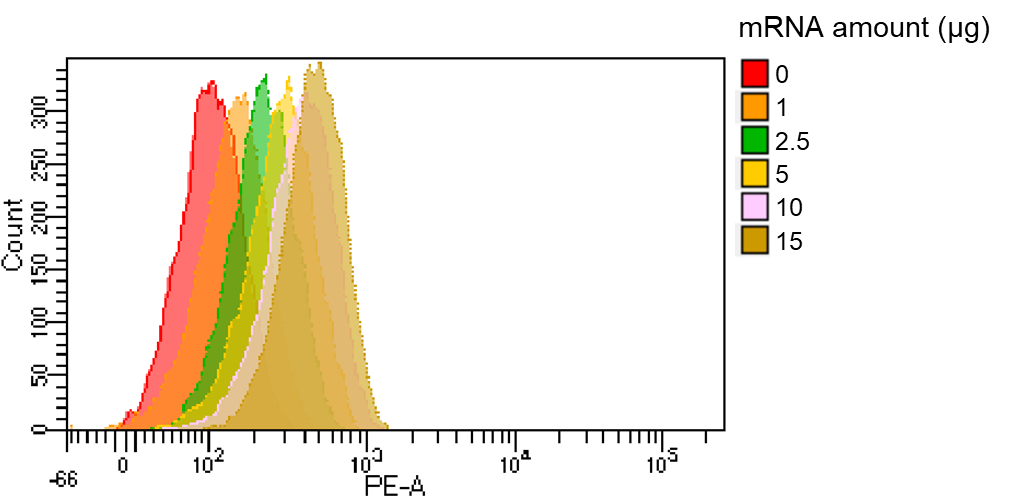


**Figure S2. tdTomato fluorescent protein expression after mRNA-LNP transfection.**

Flow cytometry analysis of HEK293FT cells treated with mRNA/LNPs (1, 2.5, 5, 10, and 15 µg tdTomato mRNA).


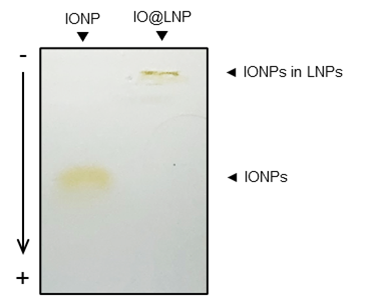


**Figure S3. Electrophoretic image of IONPs and IO@LNPs.**

The electrophoretic mobility of IO@LNPs compared with that of IONPs revealed that the negatively charged IONPs were encapsulated into positively charged LNPs in the mixture of IONPs and empty LNPs.

**
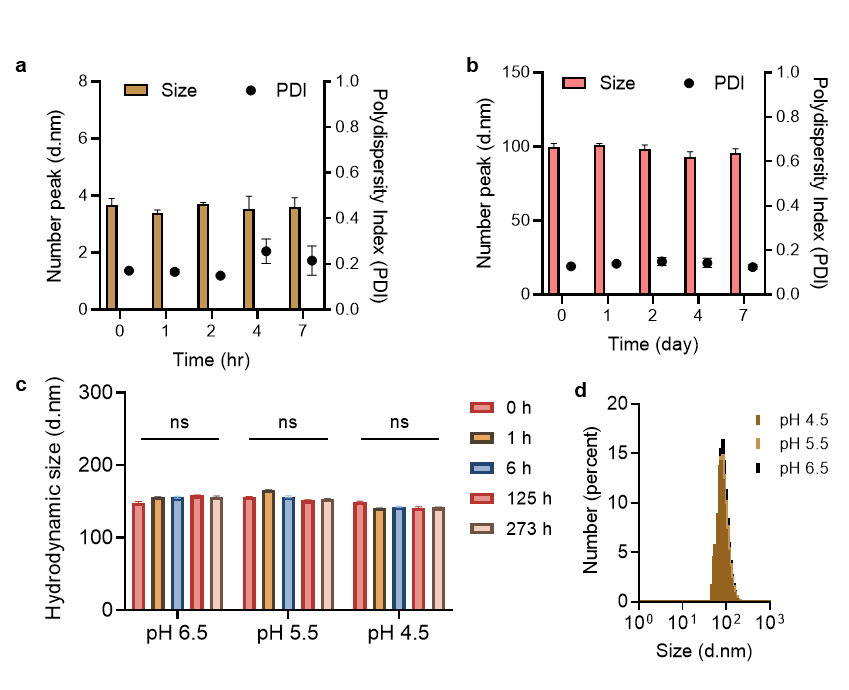
**

**Figure S4. Stability of IONPs and IO@LNPs.**

(a, b) IONP (a) and IO@LNP (b) stability based on size (diameter) distribution and PDI in PBS according to time as measured using DLS. (c) Stable hydrodynamic sizes of IO@LNPs under acidic conditions (25 mM acetate buffer) according to time (0, 1, 6, 125 and 273 h). (d) Size distribution of IO@LNP at 273 h time-point. All data presented as mean ± SEM, 3≤n≤5, *P*-values are calculated using ANOVA-test

**
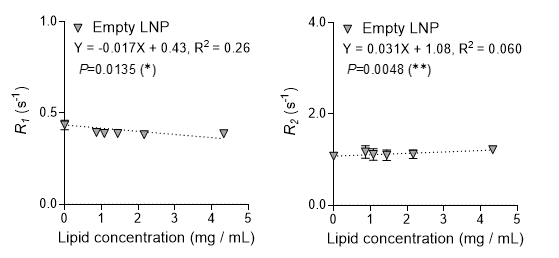
**

**Figure S5. MRI phantom test of empty LNPs.**

The lipids that constitute the LNPs exhibited marginal signals in *r*_1_ and *r*_2_. All samples were dissolved in saline. Data presented as mean ± SEM, n=3, *P*-values are calculated using F-test, * *P*<0.05, ** *P*<0.01.


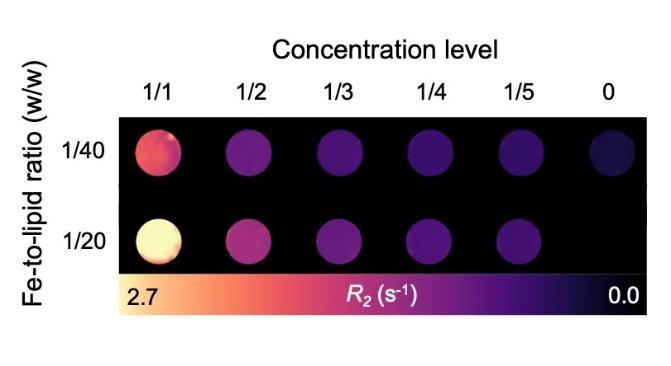


**Figure S6. *R*_2_ map of IO@LNPs with different Fe concentrations.**

*R*_2_ map image of IO@LNPs according to the Fe-to-lipid ratio (1/40 and 1/20 w/w). The samples were prepared by serial dilution from 1/1 (0.058 mg/mL Fe and 2.2 mg/mL lipid for 1/40 w/w; 0.11 mg/mL Fe and 2.2 mg/mL lipid for 1/20 w/w) to 1/5. All samples were dissolved in saline. Saline samples were set as 0. Interestingly, the *R*_2_ per Fe concentration (*r*_2_) did not significantly depend on the Fe-to-lipid ratio (*r*_2_ = 26.3 for 1/40 w/w and *r*_2_ = 27.3 for 1/20 w/w, respectively).


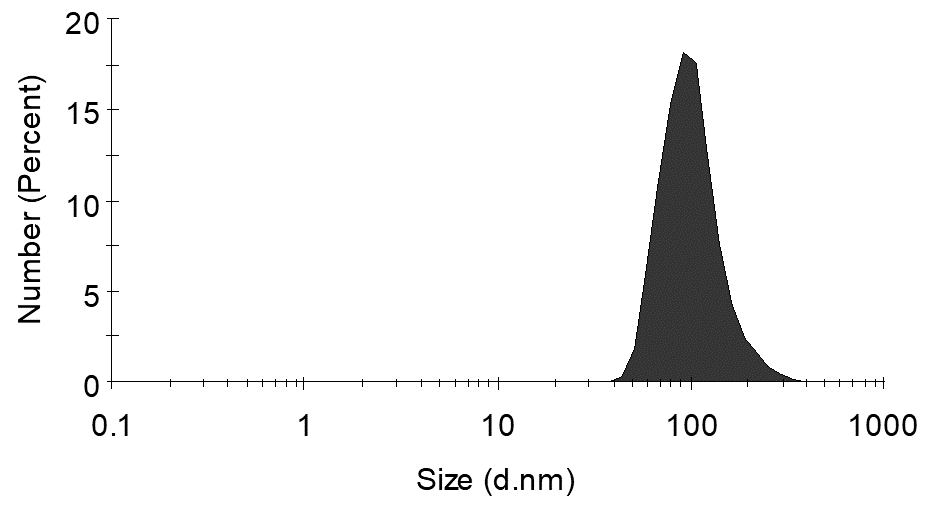


**Figure S7. Size distribution of IO@LNP/DiD.**

To evaluate their intracellular uptake, IO@LNPs were labeled with lipophilic fluorescence (DiD) dyes (See Methods). IO@LNP/DiD exhibited a hydrodynamic size of 105.2 ± 41.21 nm and PDI of 0.155. LNP, lipid nanoparticle; IO, iron oxide; PDI, polydispersity index.

**
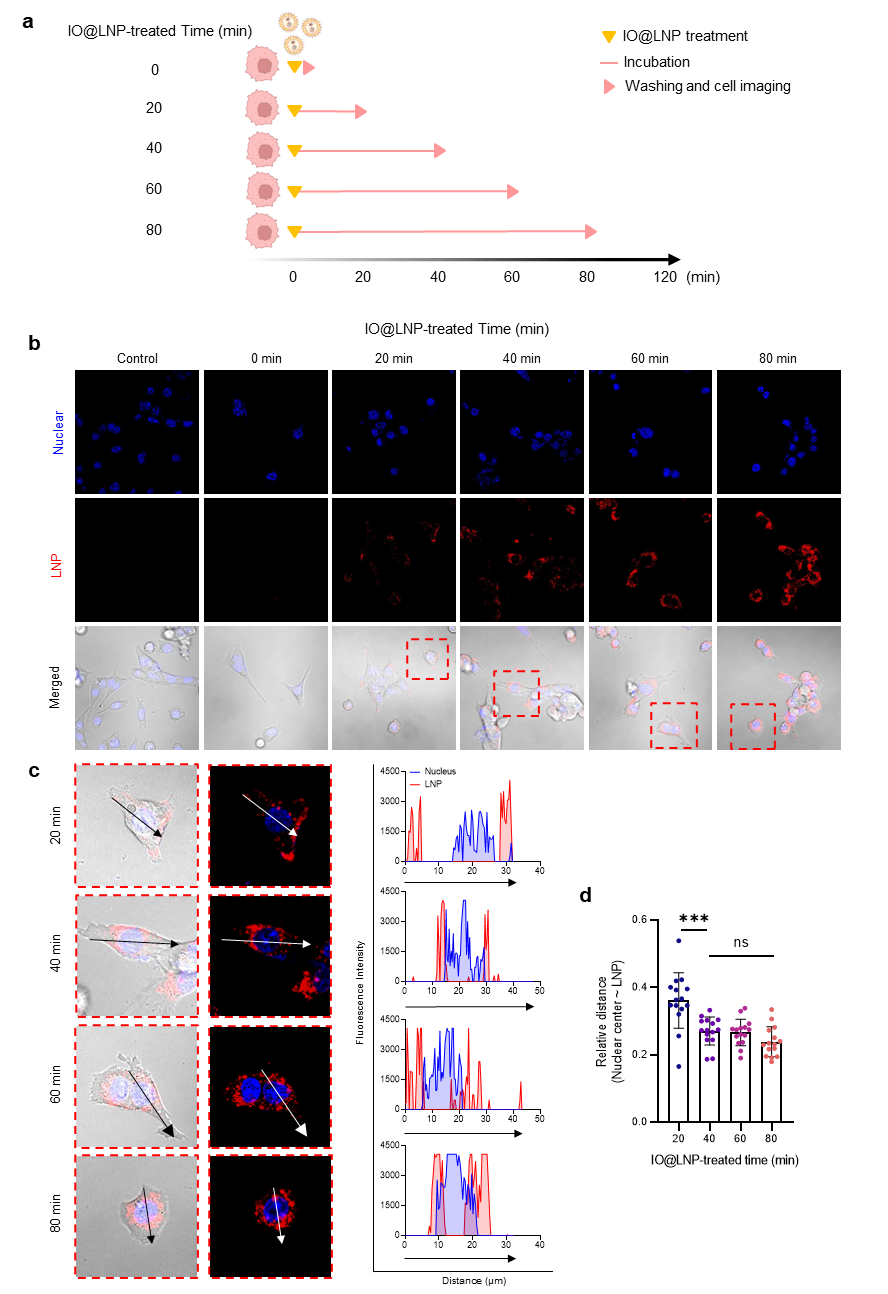
**

**Figure S8.** **CLSM images of intracellular IO@LNPs.**

(a) Experimental schedule for determining the timing for observable endosomal escape of IO@LNPs. Cells were incubated with IO@LNP/DiD (yellow), washed after different incubation durations (pink), and then imaged using confocal laser scanning microscopy (CLSM). (b) Fluorescence images showing intracellular uptake of IO@LNPs. Nuclei were stained with Hoechst 33342 (blue), and the intracellular localization of LNPs was demonstrated using DiD (red) staining. (c) The major axis of each cell was defined by a line passing through the center of the nucleus based on cross-sectional scans. In cases where the cell boundary was not clearly identifiable, a long axial reference line was manually drawn. (d) For each cell (n=15), the distance between the nuclear center and each LNP pixel on the line was normalized to the length of the line. The images showed the gradual internalization of IO@LNPs inside the cytoplasm. From 40 min after the start of incubation, LNPs were distributed in the cytoplasm, implying progressing endosomal escape. Data presented as mean ± SEM , n=15, *P*-values are calculated using ANOVA-test, *** *P*<0.001.


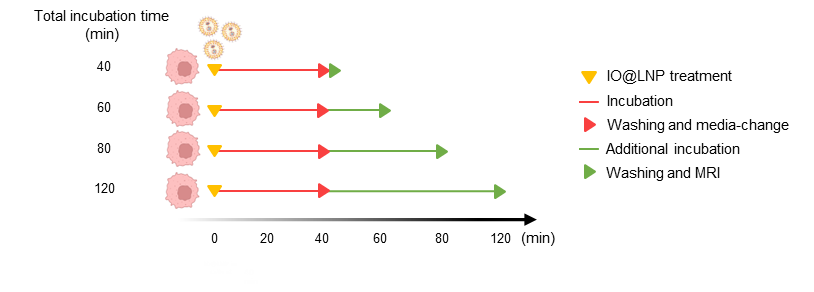


**Figure S9. Preparation of IO@LNP-treated cells for in vitro MRI.**

4T1 cells were treated with IO@LNPs for 40 min. After removing the remaining nanoparticles from the media, cells were incubated for additional time intervals (0, 20, 40, and 80 min). For each experimental group (n=3), cells were collected into pellets, fixed individually, and positioned appropriately for MR signal acquisition.

**
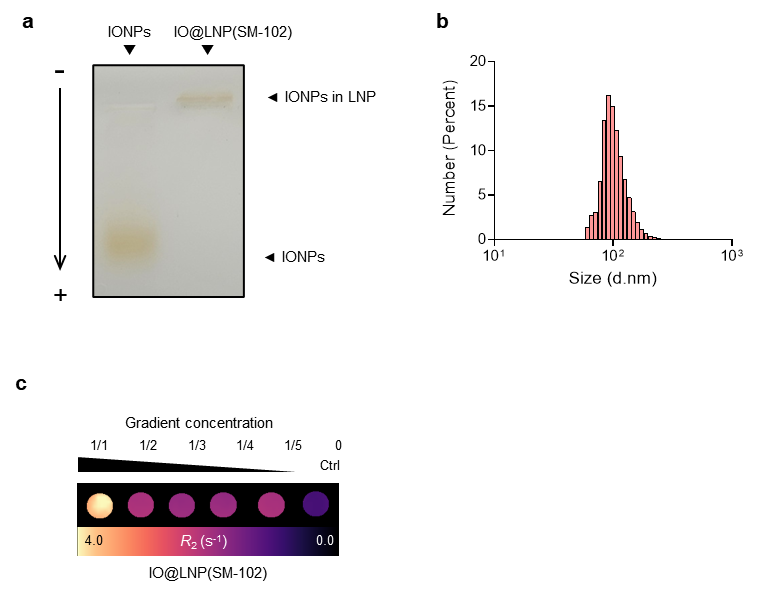
**

**Figure S10. Characterization of IO@LNPs formulated with SM-102.**

(a) An electrophoresis image of IONPs and IO@LNP formulated with SM-102 (IO@LNP(SM-102)). (b) DLS of IO@LNP(SM-102). (c) MRI phantom map.

**
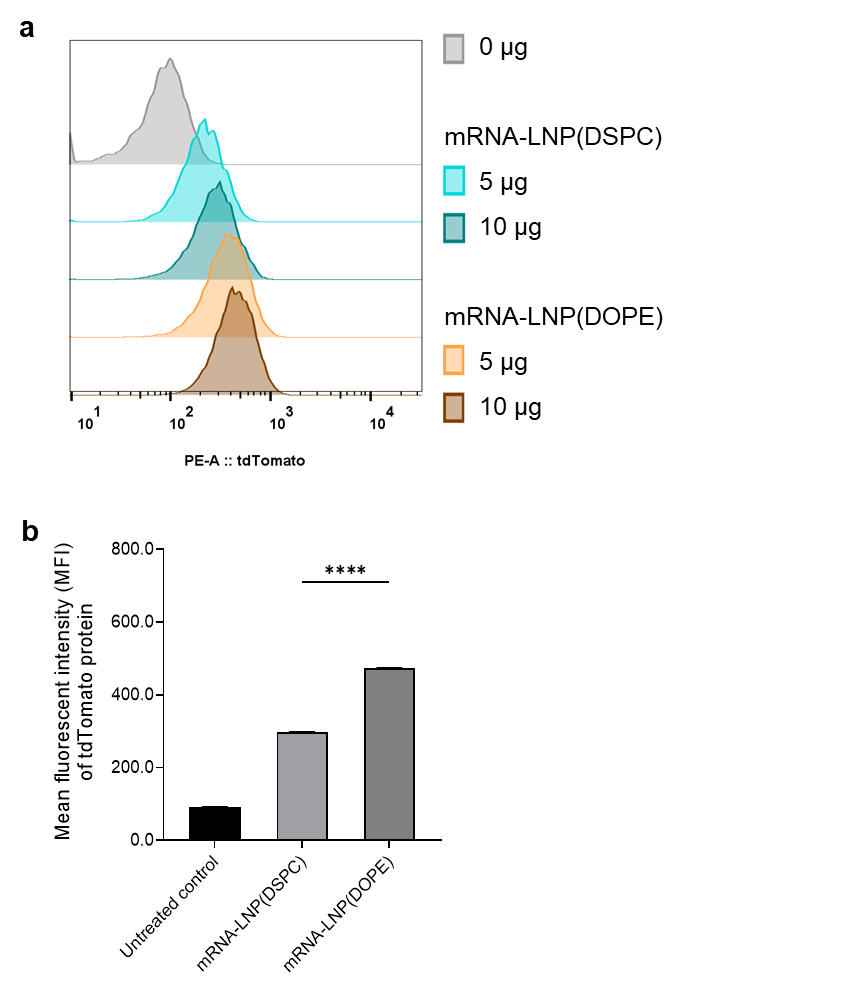
**

**Figure S11. Comparison of protein expression of mRNA delivered by different LNPs.**

(a) Flow cytometric analysis of HEK293FT cells treated with two types of mRNA/LNPs formulated with different structural helper phospholipids (DSPC or DOPE), respectively. (b) Mean fluorescence intensity of the transfected cells with tdTomato mRNA-LNP (DSPC or DOPE). Data presented as mean ± SEM, n=15, *P*-values are calculated using ANOVA-test, **** *P*<0.0001.


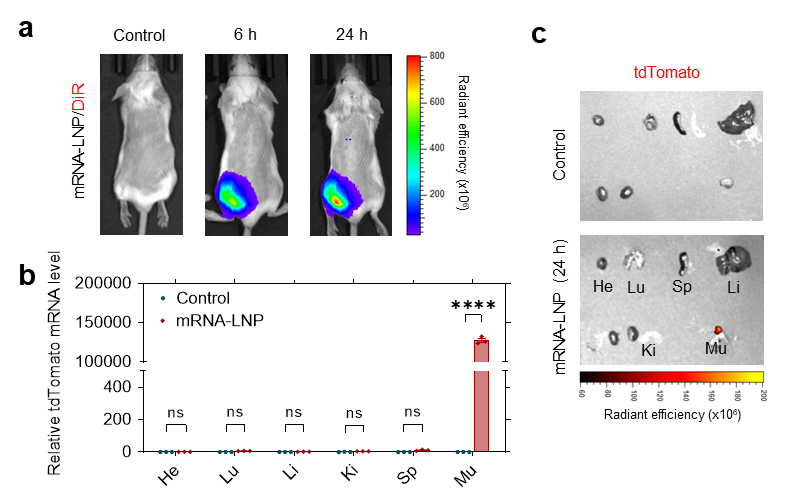


**Figure S12. In vivo delivery of mRNA-LNP**

(a) Representative IVIS spectrum images of Balb/c mouse intramuscularly injected with tdTomato mRNA-LNP/DiR. The DiR fluorescence images were acquired at 0, 6, 24 hours after injection. (b) Relative tdTomato mRNA levels in various organs 24 hours after injection. mRNAs delivered by LNP to each tissue were quantified using qPCR (Real-time PCR). (c) Representative tdTomato protein expression images. He, heart; Lu, lung; Li, liver; Ki, kidneys; Sp, spleen; Mu, muscle. Data presented as mean ± SEM, n=3, *P*-values are calculated using ANOVA-test, **** *P*<0.0001.

**
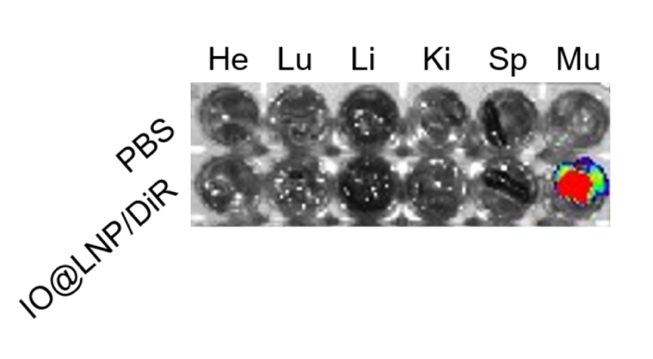
**

**Figure S13. Organ distribution of IO@LNPs.**

Balb/c mice injected with IO@LNP/DiR were euthanized, and their heart (He), lungs (Lu), liver (Li), kidneys (Ki), spleen (Sp), and muscles (Mu) were imaged using IVIS. Excluding the muscles, no fluorescent signals were observed in the other organs, including those of PBS-injected mice. PBS, phosphate buffer saline.

**
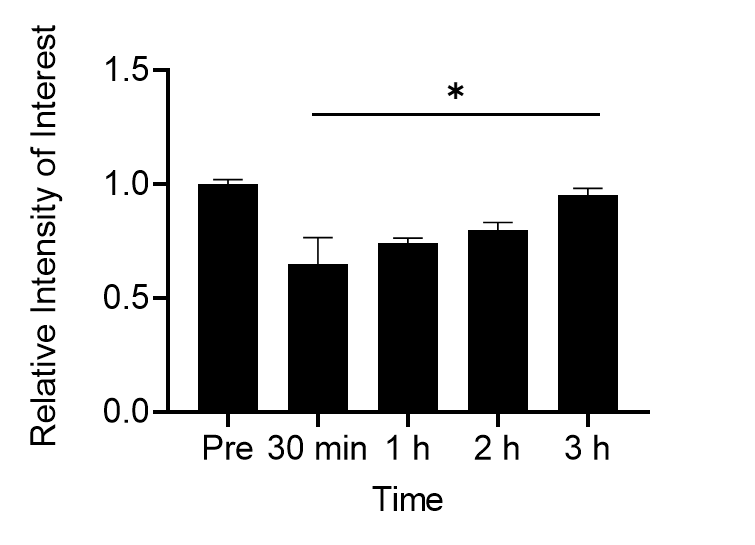
**

**Figure S14. The relative image intensity of MR contrast effect by IO@LNP derived from in vivo *T*_2_-weighted MR images.**

After multiple acquisitions of *T*_2_-weighted images of mice (N= 3), the relative intensity of dark regions induced by IO@LNPs was determined by comparing the image intensity of tissues exhibiting an MR contrast effect to those that did not in the corresponding tissues. Consequently, the MR contrast effect of loaded IONPs was the strongest immediately after injection and diminished rapidly within a few hours. A one-way ANOVA performed for 30 min, 1 h, 2 h, and 3 h showed that the time factor was statistically significant. Data presented as mean ± SEM, n=3, *P*-values are calculated using ANOVA-test, * *P*<0.05.

**
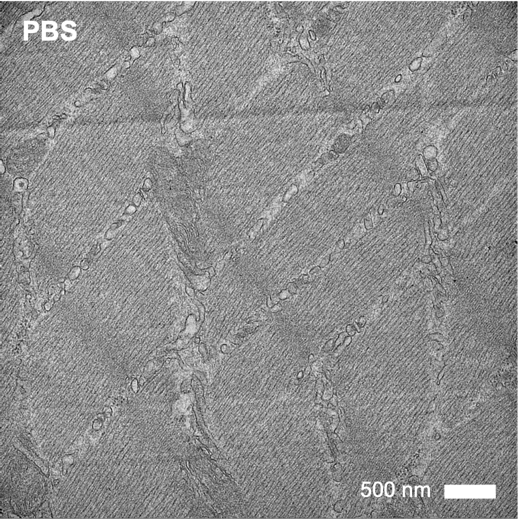
**

**Figure S15. A representative Bio-TEM image of a PBS-treated sample.**

**Table S1. tdTomato mRNA**


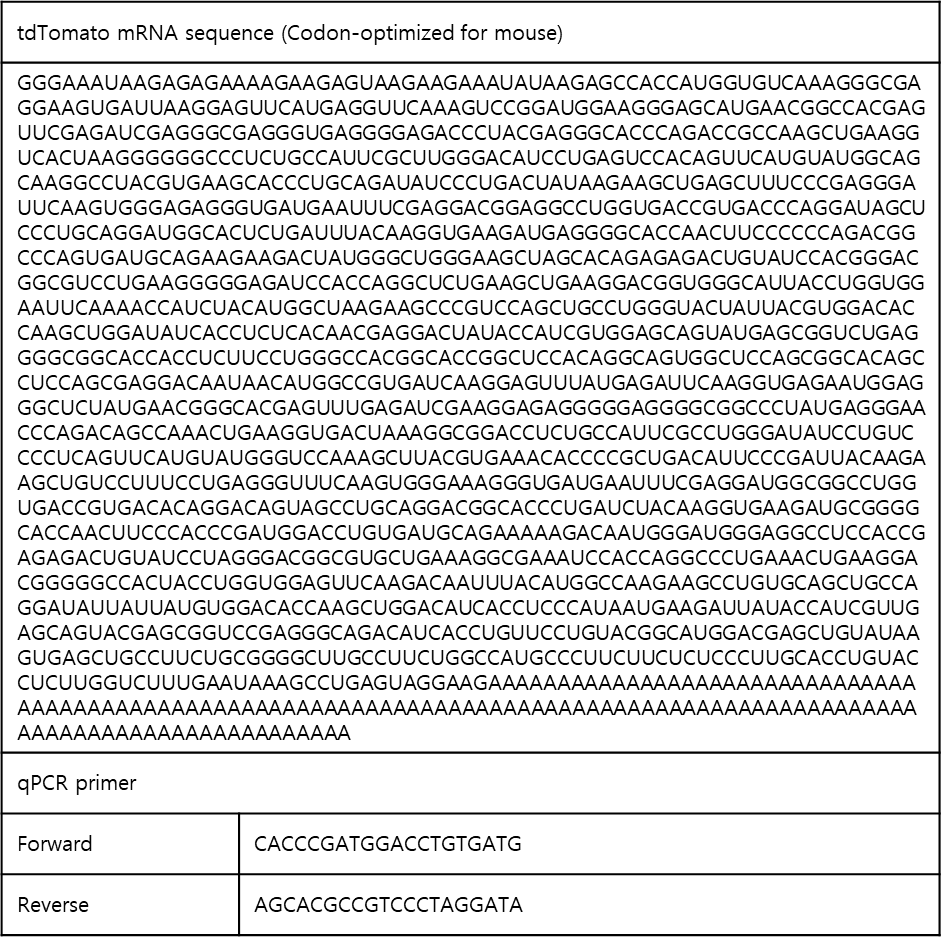


**Table S2. MRI sequence parameters corresponding to the data in Fig. 2a, b.**

|  | ***R*_1_ map** | ***R*_2_ map** |
| --- | --- | --- |
| **Sequence** | FLASH | MEMS |
| **FOV (mm)** | 30 × 52.5 | 35 × 52.5 |
| **Matrix** | 128 × 128 | 128 × 128 |
| **Slice Thickness (mm)** | 2.0 | 2.0 |
| **TR (ms)** | 8 | 5000 |
| **TE (ms)** | 0 | 9–2700 |
| **TI (ms)** | 20–4804 | - |
| **Average** | 1 | 2 |
| **Echo** | 300 | 300 |
| **Fat Saturation** | No | No |
| **Scan Time (min)** | 16 | 22 |

MRI, magnetic resonance imaging; FOV, field of view; TR, repetition time; TE, echo time; TI, inversion time

**Table S3. MRI sequence parameters corresponding to the data in Fig. 2c, d.**

|  | ***R*_1_ map** | ***R*_2_ map** |
| --- | --- | --- |
| **Sequence** | FLASH | MEMS |
| **FOV (mm)** | 30 × 52.5 | 35 × 52.5 |
| **Matrix** | 128 × 128 | 128 × 128 |
| **Slice Thickness (mm)** | 0.8 | 0.8 |
| **TR (ms)** | 8 | 5000 |
| **TE (ms)** | 0 | 9–2700 |
| **TI (ms)** | 28–4812 | - |
| **Average** | 1 | 2 |
| **Echo** | 300 | 300 |
| **Fat Saturation** | No | No |
| **Scan Time (min)** | 16 | 21 |

MRI, magnetic resonance imaging; FOV, field of view; TR, repetition time; TE, echo time; TI, inversion time

**Table S4. MRI sequence parameters corresponding to the data in Supplementary Fig. 6.**

|  | ***R*_2_ map** |
| --- | --- |
| **Sequence** | MEMS |
| **FOV (mm)** | 65 |
| **Matrix** | 128 × 128 |
| **Slice Thickness (mm)** | 2.0 |
| **TR (ms)** | 5000 |
| **TE (ms)** | 9–2700 |
| **TI (ms)** | - |
| **Average** | 2 |
| **Echo** | 300 |
| **Fat Saturation** | No |
| **Scan Time (min)** | 22 |

MRI, magnetic resonance imaging; FOV, field of view; TR, repetition time; TE, echo time; TI, inversion time

**Table S5. MRI sequence parameters corresponding to the data in Fig. 3.**

|  | ***R*_1_ map** | ***R*_2_ map** |
| --- | --- | --- |
| **Sequence** | FLASH | MEMS |
| **FOV (mm)** | 40 × 65 | 40 × 65 |
| **Matrix** | 128 × 128 | 128 × 128 |
| **Slice Thickness (mm)** | 0.5 | 0.5 |
| **TR (ms)** | 8 | 5000 |
| **TE (ms)** | 0 | 9–2700 |
| **TI (ms)** | 28–4804 | - |
| **Average** | 1 | 2 |
| **Echo** | 300 | 300 |
| **Fat Saturation** | No | No |
| **Scan Time (min)** | 16 | 22 |

MRI, magnetic resonance imaging; FOV, field of view; TR, repetition time; TE, echo time; TI, inversion time

**Supplementary references**

[1] Alipour, Z. Soran-Erdem, M. Utkur, et al., “A new class of cubic SPIONs as a dual-mode T1 and T2 contrast agent for MRI,**”***Magnetic Resonance Imaging*49 (2018): 16.
[https://doi.org/10.1016/j.mri.2017.09.013](https://doi.org/10.1016/j.mri.2017.09.013%20)

[2] Zhang, L. Liu, H. Chen, et al., “Surface impact on nanoparticle-based magnetic resonance imaging contrast agents,**”***Theranostics* 8 (2018): 2521.
<https://doi.org/10.7150/thno.23789>

[3] Huang, L. Bu, J. Xie, et al., “Effects of nanoparticle size on cellular uptake and liver MRI with polyvinylpyrrolidone-coated iron oxide nanoparticles,” *ACS Nano* 4 (2010): 7151.
<https://doi.org/10.1021/nn101643u>

[4] Hu, P. Huang, Y. Wang, et al., “Synergistic Combination Chemotherapy of Camptothecin and Floxuridine through Self-Assembly of Amphiphilic Drug–Drug Conjugate,” *Bioconjugate Chemistry* 26 (2015): 2497.
<https://doi.org/10.1021/acs.bioconjchem.5b00513>
